# Links Between Obesity-Induced Brain Insulin Resistance, Brain Mitochondrial Dysfunction, and Dementia

**DOI:** 10.3389/fendo.2018.00496

**Published:** 2018-08-31

**Authors:** Jirapas Sripetchwandee, Nipon Chattipakorn, Siriporn C. Chattipakorn

**Affiliations:** ^1^Neurophysiology Unit, Cardiac Electrophysiology Research and Training Center, Faculty of Medicine, Chiang Mai University, Chiang Mai, Thailand; ^2^Cardiac Electrophysiology Unit, Department of Physiology, Faculty of Medicine, Chiang Mai University, Chiang Mai, Thailand; ^3^Department of Oral Biology and Diagnostic Sciences, Faculty of Dentistry, Chiang Mai University, Chiang Mai, Thailand

**Keywords:** obese-insulin resistance, oxidative stress, inflammation, brain mitochondria, cognitive decline

## Abstract

It is widely recognized that obesity and associated metabolic changes are considered a risk factor to age-associated cognitive decline. Inflammation and increased oxidative stress in peripheral areas, following obesity, are patently the major contributory factors to the degree of the severity of brain insulin resistance as well as the progression of cognitive impairment in the obese condition. Numerous studies have demonstrated that the alterations in brain mitochondria, including both functional and morphological changes, occurred following obesity. Several studies also suggested that brain mitochondrial dysfunction may be one of underlying mechanism contributing to brain insulin resistance and cognitive impairment in the obese condition. Thus, this review aimed to comprehensively summarize and discuss the current evidence from various *in vitro, in vivo*, and clinical studies that are associated with obesity, brain insulin resistance, brain mitochondrial dysfunction, and cognition. Contradictory findings and the mechanistic insights about the roles of obesity, brain insulin resistance, and brain mitochondrial dysfunction on cognition are also presented and discussed. In addition, the potential therapies for obese-insulin resistance are reported as the therapeutic strategies which exert the neuroprotective effects in the obese-insulin resistant condition.

## Introduction

Obesity has been of interest in several fields of medical research. It has been demonstrated that obesity can lead to the development of several complications, including cardiovascular diseases, diabetes, and neurodegeneration (1, 2). Several reports from both *in vivo* and clinical studies also showed that obesity is associated with the development of cognitive impairment by several proposed mechanisms including the impairment of leptin signaling and the induction of Alzheimer's-like pathologies which include β-amyloid accumulation and hyperphosphorylation of tau protein (1). Another pathological condition that commonly occurs following obesity (3) is peripheral insulin resistance. This is a pathologic state in which target tissues cannot respond to insulin at the physiological level, leading to the development of hyperinsulinemia with euglycemia. Hyperinsulinemia can disturb the physiological function of several vital organs via the impairment of insulin signaling and the disturbance of intracellular signaling transduction.

The brain is one of the vital organs that can be affected as a result of peripheral insulin resistance. Several previous studies from our group and others have demonstrated that obesity not only induces peripheral insulin resistance, but can also lead to the development of brain insulin resistance, as shown by an impairment of insulin-induced long-term depression (LTD) and a reduction in the activation of brain insulin signaling pathway (4–19). One possible explanation for the occurrence of brain insulin resistance due to peripheral insulin resistance may be the production of ceramide from high lipid generation in the liver (20, 21). Ceramide, a compound of sphingosine and a fatty acid, can enter circulation and cross the blood-brain barrier (BBB). Once in the brain, ceramide can induce brain oxidative stress, brain inflammation, and brain insulin resistance, leading to neurodegeneration (22, 23).

**Figure 1:**
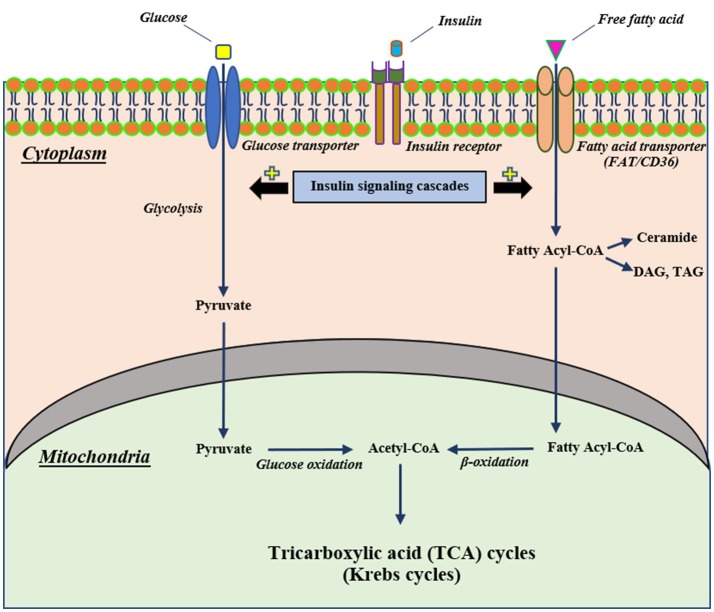
The proposed mechanism of cellular insulin signaling on glucose and fatty acid metabolisms. Extracellular insulin can bind with its receptor, resulting in stimulating insulin signaling cascades. Stimulation of insulin signaling cascades can activate cellular glucose uptake through glucose transporters that intracellular glucose further can be converted to pyruvate by glycolysis and subsequently pass into the mitochondria to change to be Acetyl-CoA for the Krebs cycle. In addition to glucose metabolism, an activation of an insulin signaling cascade can also induce intracellular uptake of free fatty acid via a fatty acid transporter (FAT/CD36) and this free fatty acid can convert to fatty acyl-CoA that translocates to mitochondria and changes to Acetyl-CoA as well as glucose metabolism. CD36, cluster of differentiation-36; DAG, diacylglycerol; FAT, fatty acid translocase; TAG, triacylglycerol.

Mitochondria are the vital organelles that provide cellular energy. They play a pivotal role in insulin signaling (24). Normally, insulin binds with its receptor, mediating the activation of cellular glucose uptake through glucose transporters. Following uptake, glucose is converted to pyruvate by the glycolytic process and these pyruvates are then converted to Acetyl-CoA, a substrate of the Krebs cycle, by glucose oxidation (25, 26). In addition, insulin stimulates the uptake of cellular fatty acids into the cells and the fatty acids are further converted to fatty acyl-CoA (25). Fatty acyl-CoA can either be converted into several lipid products, including diacylglycerol (DAG), triacylglycerol (TAG) and ceramide or be directly transported to mitochondria to induce mitochondrial β-oxidation, resulting in the production of acetyl-CoA for the Krebs cycle (25, 26). A diagram illustrating the association between insulin signaling, glycolysis and beta oxidation is summarized in Figure 1.

Previous studies reported that mitochondrial dysfunction has been related to the development of insulin resistance (26, 27). Interestingly, it has been shown that brain mitochondrial dysfunction, as indicated by the overproduction of mitochondrial reactive oxygen species (ROS), mitochondrial depolarization and mitochondrial swelling, has occurred in association with brain insulin resistance and all of these events could lead to the development of cognitive decline and Alzheimer's disease (6, 8, 11, 13–15, 19, 28–31).

Several studies have indicated a relationship between insulin resistance and mitochondrial dysfunction in cognitive-impaired rats (32, 33). Barhwal and colleagues demonstrated that an increased activation of insulin receptor subunit A (IRA) phosphorylation, subsequently stimulating the α subunit of AMP-activated protein kinase (AMPK), leading to improved brain mitochondrial biogenesis (32). Furthermore, it has been demonstrated that intranasal insulin treatment restores cognitive function in methamphetamine-induced cognitive impairment by improving brain insulin signaling via the PI3K/Akt/GSK3β pathway and improving brain mitochondrial function via key-regulatory genes related to mitochondrial biogenesis (33). Beirami and colleagues found that insulin treatment could improve insulin signaling, particularly in the PI3K/Akt/GSK3β pathway and also increase key-regulatory genes related to mitochondrial biogenesis (33). Our previous reports also demonstrated that peripheral insulin resistance develops before impaired cognition in obese-insulin resistant rat model (4, 6, 28). Taken together, all of these findings suggested that (1) The stimulation of insulin receptor plays the important roles in improving brain mitochondrial biogenesis and preserving cognitive function and (2) insulin resistance is associated with mitochondrial dysfunction and the cognitive impairment.

However, the association between obesity and brain insulin resistance, brain mitochondrial function and cognition are still not clearly understood. In this review, the current evidence regarding the associations among obesity, brain insulin resistance, brain mitochondrial dysfunction, and cognition are comprehensively summarized in this review. In addition, controversial findings in these association are presented and discussed.

## Obesity induced peripheral insulin resistance and metabolic disturbance via the induction of inflammation and increased oxidative stress

Obesity can induce peripheral insulin resistance which is an impairment of cellular insulin signaling via increased inflammatory cytokines, oxidative stress, and mitochondrial dysfunction of several tissues and organs, including adipose tissue, skeletal muscle, and the liver (34–36). Adipose tissue has commonly been acknowledged to be primarily associated with insulin resistance during obesity (37, 38). In addition to white and brown adipose tissue, beige adipose tissue also plays a possible role in the development of peripheral insulin resistance since it has ability to accumulate energy as white-type and produce heat as brown-type (39, 40).

Under peripheral insulin resistant condition, the overproduction of free fatty acids (FFAs) was occurred, resulting in a release of pro-inflammatory cytokines into the blood circulation. These cytokines can further activate several types of serine kinase such as IκB kinase (IKK) and c-Jun N-terminal kinase (JNK) (41–44). As reported previously, obese-mediated activation of these serine kinases resulted in inhibiting cellular insulin signaling by activating the phosphorylation of insulin receptor substrate-1 (IRS-1) at serine sites including serine-302 (pS302) and serine-307 (pS307), instead of its normal phosphorylated site at tyrosine residue (45–49). In addition, these cytokines activated other inflammatory-related negative regulators of IRS proteins, particularly in Suppressor of cytokine signaling (Socs) protein (50–52). This protein can bind with the phosphorylated insulin receptor (IR), consequently blocking an activation of the IRS proteins (50–52). Additionally, the Socs proteins promoted IRS proteins for ubiquitination, resulting in IRS degradation through the proteasomal complex. Those findings suggest that inflammation following obesity can lead to impaired insulin signaling or insulin resistance in the target organs.

Although numerous studies only found the association between brain mitochondrial dysfunction and brain insulin resistance in obese condition, it remains unclear whether brain mitochondrial dysfunction found in obese condition is a primary cause of brain insulin resistance. However, four previous studies suggest the possibility that mitochondrial dysfunction may be responsible for insulin resistance. The first study demonstrated that mitochondrial dysfunction induced by hyperglycemic condition impaired the AMPK-Akt pathway which is a downstream signaling cascade of the insulin signaling pathway and contributed to insulin resistance (53). Peng and colleagues suggest that mitochondrial dysfunction could lead to neuronal insulin resistance. In the second study, mice with liver-specific ablation of mitofusin-2 (Mfn2) developed glucose intolerance, enhanced hepatic gluconeogenesis as well as impaired insulin signaling in the liver and muscles (54). In the third and fourth studies, the depletion of mitochondrial DNA (mtDNA) resulted in an impaired glucose utilization and induced insulin resistance in the myotubes (55, 56). All these findings suggest that brain mitochondrial dysfunction under obesity could be the cause of brain insulin resistance.

**Figure 2:**
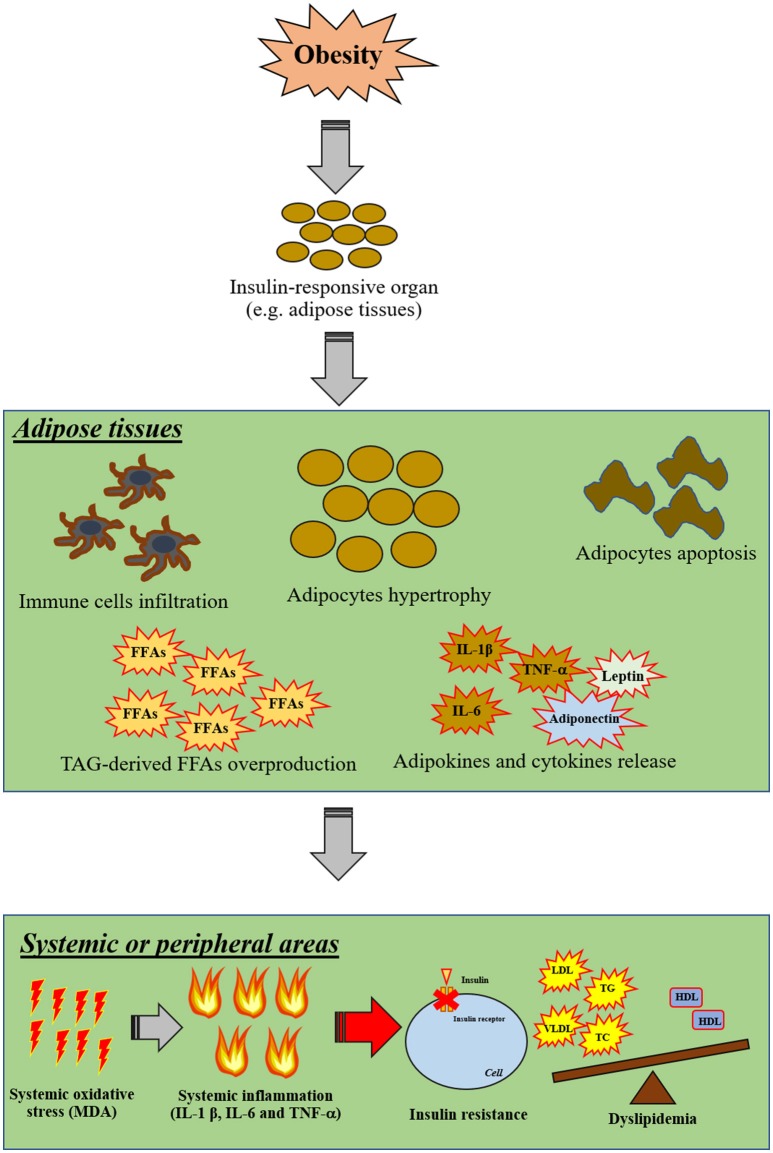
The proposed mechanisms of insulin resistance in obesity via inflammation and oxidative stress. Obesity primarily affects insulin-responsive organs particularly in adipose tissue by inducing adipocyte hypertrophy, adipocyte apoptosis, infiltration of immune cells, TAG-derived FFA overproduction and promoting the release of pro-inflammatory cytokines. Moreover, excessive FFA can also induce oxidative stress in adipose tissue. After that, cellular oxidative stress can induce systemic oxidative stress and further promote systemic inflammation. As a result, these systemic pathological conditions can impair cellular insulin signaling in other organs in association with the induction of dyslipidemia by excessive FFA production from adipose tissues. FFAs, free-fatty acids; HDL, high-density lipoprotein; IL, interleukin; LDL, low-density lipoprotein; MDA, malondialdehyde; TAG, triacylglycerol; TC, total cholesterol; TG, triglyceride; TNF, tumor necrosis factor; T2DM, type-2 diabetic mellitus; VLDL, very low-density lipoprotein; WAT, white-adipose tissue.

Excess levels of FFAs induce not only systemic inflammation, but also increase the level of oxidative stress which is the major contributory factor in the development of several co-morbidities in the obese-condition (3). Oxidative stress, involving an overproduction of free-radicals, is one of these pathological conditions. Increased oxidative stress can damage several cellular components, including mitochondria, lysosomes, endoplasmic reticulum, nuclei and DNA (57). High levels of oxidative stress can also destroy the cellular membrane, resulting in an overproduction of cytotoxic aldehyde byproducts such as malondialdehyde (MDA) and 4-hydroxylnonenal (HNE) (58). Recent studies have demonstrated that excessive FFAs in WAT also increased oxidative stress, as indicated by increasing activity of NADPH oxidase, reduced activity of antioxidative enzymes such as glutathione peroxidase (GPX), superoxide dismutase (SOD), and catalase (CAT), and decreased glutathione (GSH) synthesis (59). Excessive oxidative stress itself also induces cellular inflammation by an activation of NF-κB in association with an alteration in nuclear processes, including acetylation and deacetylation of histones. It has been shown that oxidative stress itself results in increased promotion of gene expression of pro-inflammatory mediators such as IL-1β and TNF-α in several organs (60). All those findings suggest that obesity could induce peripheral insulin resistance by the induction of inflammation and oxidative stress. The proposed mechanism around insulin resistance in cases of obesity as a possible outcome of inflammation and oxidative stress is shown in Figure 2.

## The physiological role of insulin in the brain

Under the physiological condition, insulin can be found in different brain areas, particularly in the hypothalamus, cortex and hippocampus. The level of insulin in those brain areas is higher than the plasma insulin level (61–66). In addition, Hersom and colleagues reported that the insulin level in cerebrospinal fluid has been found to be correlated with the plasma insulin level in a non-linear manner (67).

Several studies also showed that the insulin receptors (IRs) have been detected widely in different brain areas, particularly in brain areas that regulate olfaction, appetite, autonomic activity, and cognitive function (61, 68, 69). Those findings suggest that insulin in the brain not only comes from beta cells in the pancreas, but also can be synthesized from cells in the brain.

Unlike peripheral organs, several studies have shown the physiological roles of insulin in the brain are not primarily involved with the cellular glucose uptake but associated with cognitive function (70, 71). Normally, the glucose transporters (GLUT), particularly GLUT-4, play an important role in cellular glucose uptake and are regulated by insulin (72). In the brain, it has been reported that the expression of GLUT-4 is very low and GLUT-4 has not been regulated by insulin level (70). Moreover, brain GLUT-4 was in only a few areas such as the olfactory bulb, hippocampus and hypothalamus (73, 74). Along with GLUT-4, there are several types of GLUT which could require glucose uptake including GLUT-3 for neuronal glucose uptake and GLUT-1 for astrocytic glucose handling (75–77). Regarding the role of insulin in the brain, a recent study found that insulin failed to induce cellular glucose uptake into the neurons as well as the activation of insulin receptors because insulin has no effect on GLUT-4 translocation (78). Consistent with this concept, the GLUT-3-mediated neuronal glucose uptake occurs in an insulin-independent manner (79). It is possible that insulin does not play an essential role in neuronal glucose uptake, but it might have a role in the regulation of normal brain function. However, there is a recent controversial finding indicating the role of insulin-mediated glucose utilization in the brain via GLUT-4 which might affect the brain spatial working memory (80). In that study, they demonstrated that GLUT-4 inhibition, using a specific GLUT-4 inhibitor; indinavir sulfate (IND), alone did not impair neuronal glucose utilization and spatial memory in the hippocampus, whereas exogeneous insulin-mediated memory enhancement could be blocked by IND as well as impaired neuronal glucose utilization. These suggested that supra-basal insulin level might require functional GLUT-4 for neuronal glucose utilization and enhancement of brain spatial working memory (GLUT-4 dependent manner), but not for a basal insulin level which is GLUT-4 independent manner.

There is increasing evidence to show that the actions of insulin in the brain impact several aspects such as the neuromodulator, neuroprotective effects and also have a role in cognition and memory (70). Regarding the role of insulin as the neuromodulator, Kovacs and Hajnal demonstrated that insulin acts as one of the neuromodulators, as indicated by modulating GABAergic activity in the cerebellum (81). That study demonstrated that insulin exerted the GABA-dependent neuronal inhibitory effects in cerebellum, and that the effect of insulin was abolished when co-administrated with insulin receptor (IR) inhibitor. Those findings suggest that insulin in the brain plays a role as the neuromodulator. Regarding the role of insulin as the neuroprotective agent, several studies reported that insulin exerted neuroprotective effects via anti-apoptosis, β-amyloid inhibition and antioxidant agency (82–85).

Several studies regarding the benefits on brain cognition, both in *in vivo* and clinical situations have demonstrated that either peripheral or central administration of insulin showed positive effects on learning and memory (86, 87). Moreover, this effect has been shown to be related to activation via insulin receptors and downstream signaling (70, 88). The modification of learning and memory following insulin administration has been assessed by the improvement in brain synaptic plasticity, including long-term potentiation (LTP) and long-term depression (LTD) (89). Insulin could modulate neurotransmitter release at the synaptic areas particularly in the releasing of glutamate, a pivotal neurotransmitter required for the preservation of synaptic transmission (89, 90). Therefore, all those findings suggest that insulin in the brain is involved in several physiological functions, including the neuromodulation, neuroprotection, and cognition.

## Excessive free fatty acid and neuronal insulin resistance (*in vitro* studies)

As mentioned in the previous topics that insulin plays an important role in brain function, several studies demonstrated that neuronal insulin resistance can be found under obese condition (4, 6, 7, 9–14, 16–19, 28, 30, 31, 91–94). Moreover, the excessive free fatty acid (FFA) has been found under an obese condition. Growing evidence from *in vitro* studies has demonstrated the association between excessive FFA and neuronal insulin resistance. It has been shown that excessive FFA could activate the inflammation via increased release of pro-inflammatory cytokines (42, 44). Those inflammatory cytokines stimulate the activity of their downstream signaling, including the serine kinases, Ikkb, and JNK1, which subsequently inhibit the pathway of insulin receptor substrate 1 (IRS1) signaling via promoting the phosphorylation of IRS at serine sites, instead of tyrosine site (45–49). In addition, FFA can activate other inflammation-related negative regulators of IRS protein, particularly Socs proteins, and promote IRS degradation (51, 52). Those findings suggest that excessive FFA under obesity can impair insulin signaling, leading to an insulin resistant condition, via the activation of inflammation.

Several studies reported that high levels of circulating saturated free fatty acids (sFFAs) caused pathological conditions in several tissues and organs such as adipose tissue, the liver, and skeletal muscle as well as the brain, as a result of the induction of inflammation (95–100). For example, the administration of palmitate, a common sFFA, onto adipocytes, endothelial cells and myotubes led to the activation of inflammation via nuclear factor-κB (NF-κB) pathways (101–105). It has been shown that inflammation in adipose tissues triggers the release of several cytokines, resulting in aggravation of the severity of inflammation in other tissues (97, 100, 106).

A recent study established that sFFAs induced insulin resistance in the brain (107). Diaz and colleagues pointed out that the incubation of palmitate with hypothalamic cells also increased neuronal toxicity by raising ROS production, resulting in the induction of inflammation and ER stress, as well as mitochondrial dynamic impairment and reduced phosphorylation of insulin signaling protein (107).

In contrast to the previous study, Choi and colleagues demonstrated that exposure to either palmitate alone or a sFFA mixture with cultured hypothalamic neuronal cell lines induced neither neuronal inflammation nor neuronal insulin resistance (108). A possible explanation is that the type of hypothalamic neuronal cell line in Choi's study might be less sensitive to sFFA exposure, because inflammation and insulin resistance were still observed, when those cells were exposed to lipopolysaccharide (LPS). In addition, use of different types of hypothalamic cell lines might result in the different findings as shown in previous reports. All these findings from the *in vitro* models are summarized in Supplementary Table 1.

## Peripheral insulin resistance induced brain insulin resistance and brain dysfunction

A previous report has shown that peripheral insulin resistance was detected at the end of 8 weeks consumption of a HFD as characterized by an increase in body weight, an increase in visceral fat, an elevation in plasma metabolic parameters (fasting plasma glucose, fasting plasma insulin and HOMA index) and an increase in hepatic triglycerides (4). Several reports also stated that there is an association between peripheral insulin-resistance and liver-brain connection (22, 23, 109). Regarding the association between peripheral insulin resistance and brain connection, a previous study demonstrated that the development of brain insulin resistance as indicated by an impairment in both brain insulin signaling pathways and brain insulin sensitivity occurred after 12 weeks of HFD consumption (4). The possible explanation could be that peripheral insulin resistance led to increased hepatic lipid production, particularly in ceramides. Ceramide is generated from fatty acids and sphingosines (22). Ceramide is commonly known to have lipid soluble properties. Thus, it is possible to easily cross the blood-brain barrier. In addition, several studies demonstrated that ceramide induced brain insulin resistance via an impaired brain insulin signaling pathway (110), and caused neurodegeneration (22, 23, 111). Although there was no evidence showing that ceramide directly induced blood-brain barrier disruption, it is possible that a large amount of ceramide under obesity condition may be one risk factor to cause the disruption of the blood-brain barrier. Moreover, there was no direct evidence demonstrating that local insulin production in the brain can be related to the impaired insulin response.

There are a few studies reporting the timeline events of peripheral insulin resistance and the occurrence of brain insulin resistance. According to those recent studies in a rat model, chronic HFD consumption for 8 weeks only induced peripheral insulin resistance but did not initially cause brain insulin resistance. However, the extension of HFD consumption to 12 weeks could lead to the development of brain insulin resistance as shown by a reduction in insulin-related protein expression along with the impairment of insulin-induced long-term depression (LTD). These findings suggested that peripheral insulin resistance occurred prior to the brain insulin resistance.

In contrast to those reports, Filippi and colleagues reported that 3 days of lard oil-enriched HFD consumption in male Sprague-Dawley (SD) rats demonstrated brain insulin resistance (92). In addition, a study by Pathan and colleagues has been reported that metabolic disturbance including the increased plasma levels of glucose, insulin, total cholesterol, and triglyceride occurred in 5-weeks of HFD consumption (112). These different finding might be due to different compositions of HFD and different strains of animals that were used in their studies.

Supporting evidence shows that obese condition as a result of chronic consumption of a high-fat diet (HFD) or genetic-induced obesity and in obese-T2DM animals indicated the development of peripheral insulin resistance subsequently caused brain insulin resistance and could be associated with cognitive impairment (4, 6, 7, 9–14, 16–19, 28–31, 91–94, 113). Interestingly, a recent study also showed that maternal obesity can lead to the development of metabolic disturbance, hippocampal insulin resistance, and further altered the distribution of markers of neurogenesis, neuronal synaptic plasticity and function of the offspring (94). This suggest that maternal obesity might affect the offspring's neurocognitive outcome.

Systemic inflammation and oxidative stress following obesity could be the causes leading to disruption of the brain defense mechanistic structures, known as the blood-brain barrier (114). Bank and colleagues observed that under conditions of obese-insulin resistance, either circulating inflammation or oxidative stress decreased the expression of tight junction proteins in the BBB, resulting in an increase in permeability to the brain (114). That study suggested that the systemic pathological conditions occurring in the obese-insulin resistant condition, could induce brain pathological conditions, such as the induction of brain insulin resistance.

Several previous studies demonstrated that chronic HFD consumption induced obese-insulin resistance prior to causing brain insulin resistance, as indicated by decreasing phosphorylation of insulin receptors (IR), insulin receptor substrate (IRS) and downstream signaling, including PI3K-Akt, GSK3β, and AMPK pathways (4, 6, 7, 9–14, 16–19, 28–31, 91–94). Impaired brain insulin signaling in the obese-insulin resistant condition also resulted in a loss of inhibitory function to FoxO transcription factors, which normally modulate cellular metabolism and autophagy (115).

Several studies also demonstrated that obese-insulin resistance was caused by the impairment of mitochondrial function in the brain, as indicated by increased mitochondrial ROS production, induced mitochondrial membrane depolarization, and mitochondrial swelling, demonstrated by the observation of unfolded cristae in brain mitochondria (6, 8, 11–16, 19, 28–31, 92, 116). Decrease in the functional processes of mitochondria and reduced ATP content were observed in brain mitochondria of obese insulin resistant mice and Zucker diabetic rats (12, 116). Moreover, this mitochondrial change was observed, along with brain cell apoptosis, by the alteration in apoptotic related proteins including bax, bad and bcl-2 (10, 15, 19).

Although numerous studies found the association between brain mitochondrial dysfunction and brain insulin resistance which occurred in an obese condition, it remains unclear whether brain mitochondrial dysfunction in an obese condition is a primary cause of brain insulin resistance. According to Peng's study, hyperglycemic condition by high-glucose exposed to neurons led to mitochondrial dysfunction followed by an impaired AMPK-Akt signaling pathway, which is the downstream cascade of the insulin signaling pathway, and possibly contributing to insulin resistance. That finding suggested that mitochondrial dysfunction could lead to neuronal insulin resistance (53).

However, only one study from Peng and colleagues reported the idea that mitochondrial dysfunction is one causative factor for the development of brain insulin resistance. In contrast, several lines of evidence demonstrated that insulin can control mitochondrial function in pancreatic β-cells, cardiomyocytes and hepatocytes (117–119). In addition, the use of the anti-diabetic drug, thiazolidinediones, regulated the mitochondrial biogenesis suggesting that insulin can directly regulate mitochondria (120–122). All those findings imply that brain mitochondrial activity and brain insulin signaling have bidirectional communication to regulate normal brain function.

Regarding to the links among obesity-related insulin resistance, brain mitochondrial dysfunction and dementia, several studies reported that brain mitochondrial dysfunction has been observed in obese-insulin resistant condition with cognitive decline (6, 8, 11–16, 19, 28–31, 116). In addition, several pharmacological interventions that preserved brain mitochondrial function in obese-insulin resistant models could improve cognitive function (6, 8, 11–16, 19, 28–31). Those findings suggested that mitochondrial dysfunction and insulin resistance would be underlying mechanisms leading to cognitive decline.

Obese-insulin resistance caused by HFD consumption not only induced systemic inflammation, but also caused brain inflammation, as shown by increased brain pro-inflammatory cytokine; TNF-α and a transcriptional factor; NF-κB (13, 15, 19, 116). Furthermore, brain oxidative stress was shown by an elevation in cytotoxic aldehyde products; MDA was also found in association with the brain inflammation following obese-insulin resistance (8, 11, 13–15, 19, 28–31).

All of these findings suggested that obesity can increase both systemic inflammation and oxidative stress, and both events can lead to the development of peripheral insulin resistance as well as brain dysfunction.

Several studies in chronic HFD consumption-induced obese-insulin resistant condition showed an association between cognitive decline and obese-insulin resistance, occurring in association with impaired brain insulin signaling, brain mitochondrial dysfunction, brain apoptosis, increased brain inflammation, increased brain oxidative stress, and synaptic dysplasticity (8, 11–16, 19, 28–31, 123, 124). Cognitive decline was determined by several methods, including the Morris Water Maze (MWM) test (8, 11, 13–16, 19, 28–31), and a Novel Object Recognition (NOR) test (8, 12). Brain synaptic plasticity plays a pivotal role in the learning and memory process (125). Several studies reported synaptic dysfunction in the chronic HFD consumption-induced obese-insulin resistant condition, as indicated by a loss of the long-term potentiation (LTP) process, the reduction in synaptic proteins, or a decrease in dendritic spine density (9, 11, 13–15, 19, 31, 124, 126). In addition, Alzheimer's like pathologies, such as β-amyloid accumulation and tau-hyperphosphorylation have been found in obese-insulin resistant mice and diabetic rats (10, 16). All those findings suggest that obese-insulin resistance can lead to brain damage, resulting in cognitive decline and neurodegeneration.

According to several reports, the cafeteria diet could induce metabolic syndromes by altering metabolic profiles and causing induced hyperlipidemia, hyperinsulinemia and increased body weight. Although there are numerous studies that demonstrated that the cafeteria diet caused metabolic changes along with alterations in brain functions such as anxiety, depression, stress and impairment of cognition (127–135), there was no direct evidence indicating that the cafeteria diet induced brain insulin resistance.

In contrast to the HFD-induced obese-insulin resistant model, the genetically induced obese-insulin resistant models, such as *ob/ob* mice and Zucker diabetic fatty rats, developed peripheral insulin resistance without brain insulin resistance (116, 126). However, other brain dysfunction, including increased brain oxidative stress, brain mitochondrial dysfunction and brain synaptic dysplasticity were observed in those genetically-induced obese models. Those findings suggested that brain insulin resistance might not be the main cause of brain dysfunction in the genetically-induced obese-insulin resistant condition (116, 125).

Regarding the studies from type 2 diabetes mellitus (T2DM) animal models, the findings in these examples were similar to the previous findings from the model of HFD- induced obese-insulin resistance, in which peripheral insulin resistance occurred before the development of cognitive impairment, impaired brain insulin sensitivity, and brain mitochondrial dysfunction (16). The key findings of the effect of obese-insulin resistance on the brain are summarized in Supplementary Table 2.

## Evidence of brain mitochondrial dysfunction occurring in association with the development of cognitive impairment in the obese-insulin resistant condition

Mitochondria are commonly known as a cellular power house (136). Mitochondria play roles in the regulation of ATP production, Ca^2+^-storage, and the control of cell survival or death (137). As regards brain activity, a previous study has shown that mitochondria play a key role in brain synaptic transmission and age-associated cognitive function (138–141). That study suggested that changes in shape of mitochondria in presynaptic neurons affected the synaptic transmission. Donut-shape mitochondria has been reported as being a hallmark of mitochondrial stress (142, 143) and also may be related to a deficient working memory in an old-monkey model (140). In addition, the presence of donut-shaped mitochondria showed a correlation to the reduction of synapses as indicated by the smaller size of the active zone (140). All these findings indicated that brain mitochondria have a significant impact on cognitive function.

To support the concept that mitochondrial dysfunction could lead to cognitive decline, several studies reported the impact of brain mitochondria on cognitive function (144, 145). In an AD-mouse model, brain mitochondrial dysfunction and the accumulation of mitochondrial Aβ has been observed and these impairments depended on a degree of cognitive impairment in AD transgenic mice (144). In addition, Baek and colleagues demonstrated that an inhibition of mitochondrial fission (Drp-1) inhibitor ameliorated the synaptic depression, Aβ accumulation, and subsequently attenuated cognitive impairment in an AD-mouse model (145).

In addition, in cases of obese-insulin resistance, mitochondrial dysfunction was also observed in the brain (146). Several studies showed that brain mitochondrial dysfunction occurred in cases of HFD consumption, the genetically-induced obese-insulin resistant condition, and in cases of T2DM, indicated by the excessive production of mitochondrial ROS, mitochondrial membrane potential changes, and swollen mitochondria with evidence of unfolded cristae in mitochondria (6, 8, 11–16, 19, 28–31, 116). The reduction of ATP level in association with the malfunction of brain mitochondria, including decreased O2 consumption and CO2 production, has been found in brain tissue from rats with obese-insulin resistant condition (12, 116, 123, 126).

There was no evidence to directly indicate that brain mitochondrial dysfunction, particularly brain mitochondrial membrane potential change, led to brain insulin resistance. There was only an association between brain mitochondrial dysfunction and brain insulin resistance since most of studies demonstrated that brain mitochondrial dysfunction was promptly observed in obese-insulin resistant animals with brain insulin resistance as indicated by the reduction in brain insulin-related proteins expression and impairment of insulin-induced long-term depression in the hippocampal areas (5, 6, 8, 11, 14, 15, 19, 29–31).

Also, several studies have demonstrated that brain apoptosis occurs in association with brain mitochondrial dysfunction (10, 15, 19). One possible explanation is that cytochrome C is released following mitochondrial swelling, leading to the formation of a complex with APAF-1. These complexes become the apoptosomes which activate the caspase cascades and finally induce cell death (147). Previous studies showed that brain mitochondrial dysfunction increased the levels of pro-apoptotic proteins (bax and bad), and decreased levels of the anti-apoptotic protein (Bcl-2) were observed in brain tissue from rats with the obese-insulin resistant conditions (10, 15, 19). An increase in pro-apoptotic proteins can induce cytochrome C release, resulting in brain apoptosis (148). In addition, apoptotic-mediated neuronal death has been known to be one underlying mechanism for cognitive impairment and other neurodegenerative diseases (149).

In addition to brain mitochondrial function, mitochondrial dynamics, including fusion and fission, play a critical role in cell survival or death (150). It has been demonstrated that an imbalance in mitochondrial dynamics as well as malfunction of brain mitochondria has been associated with neurodegeneration, and cognitive impairment (151, 152). Previous studies demonstrated that the imbalance of mitochondrial dynamics as indicated by an increase in the mitochondrial fission process in association with a decrease in the mitochondrial fusion process, resulted in cell death and the development of cognitive decline (152, 153). Consistent with these reports, obese-insulin resistance led to a development in an imbalance in this dynamic process in the brain, specifically an increase in mitochondrial fission protein and a reduction in mitochondrial fusion protein (10, 92, 107).

All the findings pertaining to brain mitochondria suggested that brain mitochondrial dysfunction or an imbalance in mitochondrial dynamics in the brain would be the underlying mechanisms responsible for cognitive impairment associated with the obese- insulin resistant condition. The findings associated with brain mitochondria and obesity are summarized in Supplementary Table 2.

## Pharmacological interventions exerted peripheral benefits and neuroprotection against obese-insulin resistance

Obesity not only induces peripheral insulin resistance, but it also leads to brain insulin resistance, brain mitochondrial dysfunction, resulting in cognitive decline. However, several pharmacological interventions such as anti-diabetic drugs, hormone therapy, and alternative medicine, which exert beneficial effects on peripheral insulin sensitivity, have also been reported to provide neuroprotection in the brain. Despite medication, non-pharmacological intervention as well as subcellular targeting interventions also provide benefits in the brain under obese-insulin resistant condition. All these findings are summarized and discussed in the following section.

### Neuroprotective effects of therapeutic interventions using unsaturated free fatty acid *in vitro* studies

Recent *in vitro* study found unsaturated free fatty acids provided beneficial effects in neuronal insulin-resistance caused by saturated free fatty acid (sFFA) exposure through improving cell insulin-related signaling and mitochondrial function in association with reducing neuronal inflammation, oxidative stress, and apoptosis (107). This finding suggested that neuronal insulin resistance by sFFA induction occurred through the impairment of neuronal inulin-related signaling, mitochondrial dysfunction, and inflammation-oxidative stress and that intervention using unsaturated free fatty acids could ameliorate this neurotoxicity.

### Beneficial effects of anti-diabetic drugs on the brain in the obese-insulin resistant condition

Therapeutic interventions including anti-diabetic drugs and hormone therapy are required to relieve the deleterious effects occurring as a result of obese-insulin resistance (154, 155). Of these interventions, anti-diabetic drugs are commonly known to reduce body weight, decrease dyslipidemia, and improve insulin sensitivity in the obese-insulin resistant condition (155).

Several classes of anti-diabetic drugs, including a peroxisome proliferator-activated receptor gamma (PPARγ) agonist, biguanide, dipeptidyl peptidase-4 (DPP-4) inhibitors, and sodium-glucose co-transporter 2 (SGLT-2) inhibitors, have been used in obese-insulin resistant models. These drugs not only provided beneficial effects in attenuating metabolic disturbance, but also exerted neuroprotective effects, as indicated by improved brain insulin sensitivity, brain mitochondrial function and hippocampal synaptic plasticity, as well as reducing brain inflammation, brain oxidative stress, brain apoptosis, and dendritic spine loss. They also led to improved cognitive function (6, 17, 19, 28–31, 112, 113, 124, 156, 157).

The neuroprotective effects of pharmacological interventions particularly anti-diabetic drugs and hormone therapy could possibly be due to direct effects in the brain since some types of these drugs can pass the blood-brain barrier (158, 159) and improve the periphery.

### Beneficial effects of hormone therapy in the brain in the obese insulin-resistant condition

Not only the use of anti-diabetic drugs, but also the addition of incretin hormone therapy is needed in some cases to ameliorate the obese-insulin resistant condition (160). Incretin hormones are a group of metabolic hormones that play a role in decreased blood glucose levels via stimulating insulin secretion in response to meals (161). Incretin hormones include glucagon-like peptide-1 (GLP-1) and glucose-dependent insulinotropic polypeptide (GIP) (162). Both GLP-1 and GIP are secreted from enteroendocrine cells in the intestinal epithelium. Recently, incretin hormones have been considered as a potential intervention for diabetic therapies since they exert blood-glucose lowering effects (161). In addition, several previous studies reported that incretin hormones not only reduced blood glucose levels, but also exerted other beneficial effects such as anti-oxidant, anti-inflammation, anti-apoptotic effects and an enhancement of cell proliferation (163–165).

However, the use of incretin hormone therapy showed controversial effects as regards neuroprotection in conditions of obese-insulin resistance (123, 126, 166, 167). Examples of the findings include: (1) Use of an incretin mimetic drug, exendin-4, resulted in neuroprotective effects by improving hippocampal synaptic plasticity and cognitive function along with improvement of metabolic parameters in an obese-insulin resistant condition (166). (2) Another incretin mimetic drug, liraglutide, in cases of either HFD or genetically induced obese insulin resistance demonstrated that the improvement in metabolic parameters along with a preservation in the neurogenesis and neuronal survival resulted in improved hippocampal synaptic plasticity and cognitive function (126, 167). (3) Lixisenatide, known as GLP-1 receptor agonist, could improve cognitive performance by attnueating peripheral insulin resistance and enhancing cells proliferation in brain of HFD-fed animal model by Lennox et al. (168). (4) However, use of incretin metabolites [GLP-1(9-36), GIP (3-42) and exendin (9-39)] did not lead to the attenuation of either metabolic or brain parameters including hippocampal synaptic plasticity, brain locomotor activity and cognitive function in the HFD-fed mice model (123). The possible explanation for the differences could be due to the differences in pharmacokinetic structures between incretin metabolites and incretin mimetic drugs. This possibility requires further investigation to explore these controversial findings.

In addition, the administration of fibroblast growth factor-21, a starvation hormone, also exerted neuroprotection in the obese-insulin resistant condition (15, 113). To support these findings, FGF21 administration has been reported to improve peripheral parameters including (1) increased an energy expenditure, resulting in body weight reduction (169, 170), (2) improved peripheral insulin sensitivity through an increased adipocyte glucose uptake rate, increased insulin synthesis and decreased hepatic gluconeogenesis (171–174), (3) exerted anti-inflammatory effect (175), and (4) increased adiponectin biosynthesis (176, 177). In addition, FGF21 has also demonstrated the neuroprotective effects by several underlying mechanisms including (1) improving mitochondrial biogenesis and function, by increasing mitochondrial respiratory capacity and mitochondrial anti-oxidant levels (178) and (2) decreasing brain cell damage (179).

### Beneficial effects of alternative medicine in the brain in the obese insulin-resistant condition

Several studies have shown that other alternative interventions, including herbal extracts or recipes, also exerted neuroprotective effects in the obese-insulin resistant condition (8, 12, 13, 15, 16, 180). Interestingly, the beneficial effects of these interventions were not only indicated in the peripheral area, but also provided neuroprotective effects against cognitive impairment in the obese insulin-resistant condition (8, 12, 13, 15, 16, 180).

Herbal extracts, including cinnamon extract, garlic extract, naringin, a ZiBu PiYin recipe, Thymol and Ginsenoside Re have recently been reported as leading to improvements in brain cognition and locomotor activity in obese insulin-resistant animals with or without the improvement of metabolic parameters (8, 12, 16, 17, 23, 93).

### Beneficial effects of subcellular-targeting intervention in brain in the obese-insulin resistant condition

Interestingly, application of a N-methyl D-aspartate receptor (NMDARs) antagonist in a genetically-induced obese-model also demonstrated neuroprotective effects through rescuing dendritic spine arborization and synaptic density (180). Moreover, the inhibition of mitochondrial fission by MDIVI-1 could prevent brain insulin resistance through the attenuation of ER stress and iNOS expression (92).

### Beneficial effects of non-pharmacological intervention in brain in the obese-insulin resistant condition

In addition, non-pharmacological interventions, including energy restriction and vagus nerve stimulation, also exerted neuroprotective effects in the obese-insulin resistant condition (13, 31). Regarding obese-insulin resistant models, energy restriction attenuated metabolic disturbance and preserved dendritic spine density (31), while the vagus nerve stimulation led to attenuated peripheral insulin resistance as well as improved brain function, as indicated by improved brain insulin sensitivity/brain mitochondrial function/cognitive function, and reduced brain inflammation, /brain oxidative stress/ brain apoptosis/dendritic spine loss (13).

Taken together, all those findings from therapeutic interventions suggested that all interventions in the treatment of obese-insulin resistance not only improved the metabolic parameters, but also protected the brain against cognitive decline. All these findings are summarized in Supplementary Table 3.

According to previous reports, pharmacological interventions such as anti-diabetic drugs can attenuate cognitive impairment via the improvement of peripheral insulin sensitivity (181, 182), the provision of antioxidative effects (17, 19, 28–31, 157, 183), a decrease in inflammation and apoptosis (17, 19, 113, 157, 183), as well as the improvement of mitochondrial function in the brain (6, 19, 28–31, 113). Another factor associated with cognitive impairment is an imbalance in brain mitochondrial dynamics (92). Only one previous study has demonstrated that a modulation of brain mitochondrial dynamics attenuated neuronal insulin resistance (92). The beneficial effects of this modulation on cognitive function in obese models have not yet been investigated. Therefore, further investigation is needed to prove this mechanism.

## Conclusion

**Figure 3:**
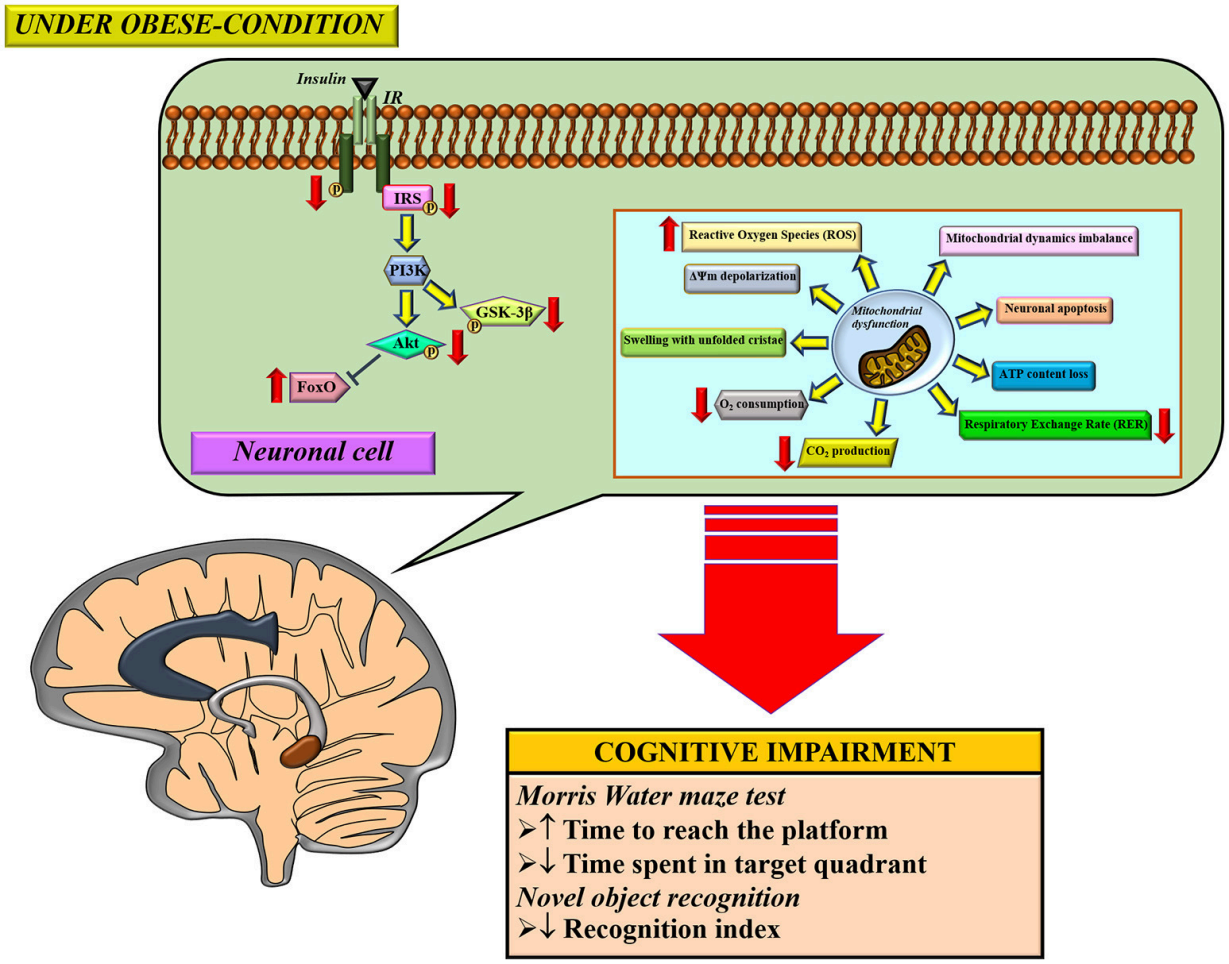
The proposed mechanisms of obese-insulin resistance and brain mitochondrial dysfunction induced brain cognitive decline. Under obese-condition, brain insulin signaling was initially impaired by a reduction of insulin receptor (IR) phosphorylation which led to further its downstream signaling cascade including decreasing phosphorylation of the insulin receptor substrates (IRS), Akt and GSK3β. On the other hand, the reduction in Akt phosphorylation subsequently decreased an inhibitory response to FoxO protein, resulting in an increased FoxO expression. In parallel with impaired brain insulin signaling pathways, brain mitochondrial dysfunction was also observed as indicated by several anomalies in function changes (increased ROS production, ΔΨm depolarization, in association with decreased O_2_ consumption, CO_2_ production and respiratory exchange rate (RER), and thus induced loss of ATP content), morphological changes (mitochondrial swelling and unfolded cristae), dynamics changes (imbalance of mitochondrial fusion and fission proteins expression), and causing neuronal apoptosis. Eventually, all these pathological changes can lead to cognitive impairment, determined by learning and memory behavior tests. ΔΨm; mitochondrial membrane potential, ATP; adenosine triphosphate, FoxO, forkhead box O; GSK-3β, glycogen synthase kinase-3-beta; IR, insulin receptor; IRS, insulin receptor substrate.

Several studies demonstrated that obese-insulin resistance contributed to cognitive impairment through several proposed mechanisms, including inflammation and oxidative stress. Brain mitochondria are also damaged as a result of an obese-insulin resistant condition with resulting cognitive decline. Therapeutic approaches for obese-insulin resistance not only had beneficial effects to the metabolic parameters, but also led to improvement in brain function, brain mitochondrial function, and cognitive function. Therefore, it is possible that mitochondria may play an important role in cognitive decline in conditions pertaining to obesity. A summary of the possible mechanisms involved in the impact of obese-insulin resistance on the brain is shown in Figure 3. Although many studies have reported an association between peripheral insulin resistance and brain pathologies such as brain insulin resistance, brain mitochondrial dysfunction, and cognitive impairment, these studies could not conclude the causative relationship between insulin resistance and brain mitochondrial dysfunction. All of these studies imply that an improvement in peripheral insulin sensitivity along with an improvement in brain mitochondrial function could preserve cognitive function under obese condition. Further investigations are needed to better understand the underlying mechanisms of cognitive impairment in obese condition. This information is necessary to provide specific molecular targets for drug development programs.

## Author contributions

Conception and design of the study: JS, NC, and SC. Analyzed data: JS, NC, and SC. Initial draft of manuscript: JS and SC. Manuscript editing: JS, NC, and SC. All authors approved the final version of the manuscript.

### Conflict of interest statement

The authors declare that the research was conducted in the absence of any commercial or financial relationships that could be construed as a potential conflict of interest.
