# Supplementary material for: Links Between Obesity-Induced Brain Insulin Resistance, Brain Mitochondrial Dysfunction, and Dementia
Source: Front Endocrinol (Lausanne). 2018 Aug 31;9:496. doi: 10.3389/fendo.2018.00496 (PMC6127253; doi:10.3389/fendo.2018.00496)
Supplement: Supplementary file 1 [file Table_1.DOC]

**Supplementary Table 1:** Effects of obesogenic condition (↑ free fatty acid) onto neuronal insulin-related signaling: *in vitro* studies

| **Study Models** | **Methods**  **Fat /dose /time** | **Major findings** | **Interpretation** | **Refs.** |
| --- | --- | --- | --- | --- |
| Hypothalamic mHypoA-CLU192 cells | - Palmitic acid/250 and 500 µM/ 6, 12, 24 and 48 hours | **Palmitic acid exposure**   - ↑ mitochondrial ROS production (only 500 µM palmitic acid) - ↓ Mfn-2, p-Akt_Ser473_ proteins expression - ↑ GRP78/BIP marker - ↔ cell death | ROS production during palmitic stimulation plays role in inflammation and ER stress, via impaired mitochondrial dynamics, possibly resulting to impact on insulin signaling pathway. | (Diaz et al., 2015) |
| Cultured hypothalamic neurons from mouse cell line GT1–7 and N43/5 | - Mixture of PA, SA, AA and BA conjugated with BSA (MFA)/100, 250 and 500 µM/ 3 and 24 hours - Palmitate /200 µM/ 1, 3, 6 and 24 hours | **MFA exposure 24 hours in**  ***GT1-7 cells***   - ↑ IκBα and IL-6 mRNA expression (only at 500 µM of MFA) with a significant cell death - ↔ p-Akt/ total-Akt ratio, IRS-1 and p-Tyrosine expression   ***N43/5 cells***   - ↔ IκBα and IL-6 mRNA expression in at any concentration   **Palmitate treatment**   - ↑ p-ERK1/2 and Chop mRNA expression - ↔ p-NF-κB mRNA expression | Saturated fatty acid exposure did not induce inflammatory signaling or insulin resistance in cultured hypothalamic neurons. | (Choi et al., 2010) |

*AA; arachidic acid, ATP; adenosine triphosphate, BA; behenic acid, BSA; bovine serum albumin, Chop; CCAAT-enhancer-binding protein homologous protein, DAG; diacylglycerol, ER; endoplasmic reticulum, ERK; Extracellular signal-regulated kinase, GRP78/BIP; 78 kDa glucose-regulated protein/binding immunoglobulin protein, IκB; I kappa B, IL-6; interleukin-6, iNOS; inducible nitric oxide synthase, IRS-1; insulin receptor substrate-1, JNK; c-Jun N-terminal kinases, NF-κB; nuclear-factor kappa B, PA; palmitic acid, PGC-1α; peroxisome proliferator-activated receptor gamma coactivator 1-alpha, ROS; reactive oxygen species*, SA; stearic acid.
