# Supplementary material for: Links Between Obesity-Induced Brain Insulin Resistance, Brain Mitochondrial Dysfunction, and Dementia
Source: Front Endocrinol (Lausanne). 2018 Aug 31;9:496. doi: 10.3389/fendo.2018.00496 (PMC6127253; doi:10.3389/fendo.2018.00496)
Supplement: Supplementary file 2 [file Table_2.DOC]

**Supplementary Table 2: Effects of obesogenic condition ( free fatty acid) onto the brain insulin resistance, brain inflammation, brain oxidative stress and cognitive function: *in vivo* studies**

| **Study Models** | **Methods** | **Major findings** | **Interpretation** | **Refs.** |
| --- | --- | --- | --- | --- |
| **1. High-fat diet induced obese-insulin resistant models** | | | | |
| Male Sprague Dawley rats | - High-fat diet (HFD, 58%E from fat) for 5 weeks | **HFD**-**fed rats**   -  body weight -  plasma glucose, TC, TG and insulin -  escape latency | HFD consumption impaired cognitive performance via metabolic disturbance. | (Pathan et al., 2008) |
| Male Swiss TO mice (6–8 weeks old) | - High-fat diet (HFD, 45%E from fat) for 8 months - Whole brain and hippocampal slices | **HFD**-**fed mice**   - body weight, average energy intake, plasma glucose, plasma insulin and AUCg -  AUCi - LTP -  RI in trial phase | HFD consumption developed peripheral insulin resistance and then leading to impair hippocampal synaptic plasticity and cognitive function. | (Gault et al., 2010) |
| Male Wistar rats | - High-fat diet (HFD, 59.28%E from fat) for 4, 8 and 12 weeks - Whole brain and hippocampal slices | **4 weeks of diet consumption in rats**   -  body weight -  plasma cortisone   **8 weeks of diet consumption in rats**   -  body weight and VF -  FPG, plasma insulin and HOMA index -  liver TG -  plasma cortisone   **12 weeks of diet consumption in rats**   -  body weight and VF -  FPG, plasma insulin, HOMA index and AUCg -  liver TG -  plasma and neuronal cortisone -  brain p-IR, p-IRS-1, total-IRS-1 and p-AktSer473 proteins expression -  insulin-induced LTD | - Peripheral insulin resistance developed at 8-week HFD-fed rats. - Elevated neuronal corticosterone level and brain insulin resistance were found at 12-week HFD consumption. | (Pratchayasakul et al., 2011b) |
| Wistar rats (both genders) | - High-fat diet (HFD, 59.28%E from fat) for 12 weeks - Whole brain and hippocampal slices | **HFD**-**fed male rats**   -  body weight and VF -  FPG, plasma insulin and HOMA index -  liver TG -  insulin-induced LTD -  brain p-AktSer473 proteins expression   **HFD**-**fed female rats**   -  body weight and VF -  FPG, plasma insulin, plasma TG and HOMA index -  liver TG -  insulin-induced LTD -  brain p-AktSer473 proteins expression | Both male and female rats fed with 12-weeks HFD consumption developed peripheral insulin resistance along with brain insulin resistance. | (Pratchayasakul et al., 2011b) |
| Male Wistar rats | - High-fat diet (HFD, 59.28%E from fat) for 12 weeks - Whole brain and hippocampal slices | **HFD-fed rats**   -  body weight and VF -  plasma TC and insulin -  AUCg­ and HOMA-index -  brain mitochondrial ROS production -  brain mitochondrial depolarization -  brain mitochondrial swelling -  brain p-AktSer473 expression -  insulin-induced LTD | Brain insulin resistance and the impairment of brain mitochondria occurred following 12 weeks HFD consumption. | (Pipatpiboon et al., 2012) |
| Male Wistar rats | - High-fat diet (HFD, 59.28%E from fat) for 12 weeks - Whole brain | **HFD**-**fed rats**   -  body weight and VF -  plasma TC and insulin -  plasma and brain MDA -  AUCg­ and HOMA-index -  brain mitochondrial ROS production -  brain mitochondrial depolarization -  brain mitochondrial swelling -  time to reach the platform -  time spent in target quadrant | Long-term HFD consumption caused peripheral insulin insensitivity, brain mitochondrial dysfunction and impaired learning behavior. | (Pintana et al., 2012) |
| Male Swiss *TO* mice (6-8 weeks old) | - High-fat diet (HFD, 45%E from fat) for 4 months - Whole brain and hippocampal slices | **HFD**-**fed mice**   -  body weight, non-FPG -  insulin to glucose ratio - impaired glucose and insulin response -  O2 consumption or CO2 production -  locomotor activity - LTP -  RI on trial phase | HFD impaired insulin sensitivity and induced brain mitochondrial dysfunction, resulting in brain dysfunction. | (Porter et al., 2012) |
| Male Wistar rats | - High-fat diet (HFD, 59.28%E from fat) for 12 weeks - Whole brain and hippocampal slices | **HFD**-**fed rats**   -  body weight and VF -  plasma total cholesterol and insulin -  plasma and brain MDA -  AUCg­ and HOMA-index -  plasma and brain GLP-1 -  k-value from IGTT -  brain mitochondrial ROS production -  brain mitochondrial depolarization -  brain mitochondrial swelling -  brain p-AktSer473, p-IR and p-IRS proteins expression -  insulin-induced LTD -  time to reach the platform -  time spent in target quadrant | Obese-insulin resistant condition led to the development of brain insulin-resistance, brain mitochondrial dysfunction, increased brain oxidative stress, and impaired cognitive function. | (Pipatpiboon et al., 2013) |
| Male Wistar rats | - High-fat diet (HFD, 59.28%E from fat) for 12 weeks - Whole brain | **HFD**-**fed rats**   -  body weight and VF -  plasma TC and insulin -  plasma and brain MDA -  AUCg­, AUCi and HOMA-index -  plasma GLP-1 and HDL -  brain mitochondrial ROS production -  brain mitochondrial depolarization -  brain mitochondrial swelling -  time to reach the platform -  time spent in target quadrant | HFD consumption induced peripheral insulin resistance and decreased brain function, and brain mitochondrial dysfunction. | (Pintana et al., 2013) |
| Male Wistar rats | - High-fat diet (HFD, 59.28%E from fat) for 12 weeks - Whole brain and hippocampal slices | **HFD**-**fed rats**   -  body weight and VF -  plasma TC, glucose, insulin and HOMA-index -  dendritic spine numbers | HFD-fed rats caused peripheral insulin resistance as well as the reduction of the density of dendritic spines in CA1 hippocampus. | (Sripetchwandee et al., 2014) |
| Male Wistar rats | - High-fat diet (HFD, 59.28%E from fat) for 12 weeks - Whole brain | **HFD**-**fed rats**   -  body weight and VF -  plasma TC and insulin -  plasma and brain MDA -  AUCg­ and HOMA-index -  brain mitochondrial ROS production -  brain mitochondrial depolarization -  brain mitochondrial swelling -  time to reach the platform -  time spent in target quadrant | Long-term HFD consumption induced peripheral insulin resistance, oxidative and brain mitochondrial dysfunction, possibly resulting brain insulin resistance. | (Pintana et al., 2014) |
| C57BL/6J mice  (8 weeks-old) | - Extreme High-fat diet (HFD) (60%E from fat) for 17 days - Moderate HFD (45%E from fat) for 8 weeks - Cerebral cortex and hippocampus | **HFD-fed mice**   -  weight gain and plasma glucose - Inhibited activation of Akt, S6 and GSK3β after ex-vivo insulin stimulation -  p-IRS1Ser616 expression -  IMPK, PSD-95 and synaptopodin expression -  spontaneous alternation in a T-maze | HFD consumption induced brain insulin resistance, deleterious effects on synaptic integrity and cognitive deficit. | (Arnold et al., 2014) |
| Male C57BL/6J (B6) mice (4 weeks old) | - High-fat diet (HFD, 60% E from fat) for 23 weeks - Whole brain | **HFD consumption**   -  BW -  FPG and insulin -  plasma AUCg during ITT -  mean brain weightandmean brain weight**/**BW ratio -  IR protein expression -  immunofluorescence of IR in hippocampus -  brain Akt and p-Akt proteins expression in nuclear fraction -  brain Foxo3a protein expression in nuclear fraction -  brain p-Akt and Foxo3a protein expression in cytosolic fraction -  brain p-Foxo3a protein expression in cytosolic fraction -  brain mRNA levels of Foxo3a and apoptotic genes (Bim, Fas-L and p27) -  brain bad-2, VDAC, HSP60 proteins expression -  brain mRNA levels of mitochondrial fission (Drp-1 and Fis-1) -  brain mRNA levels of mitochondrial fusion (OPA1 and Mfn-1) -  brain p-ERK and iNOS proteins expression -  brain SOD and H-Oxy proteins expression -  AD-related proteins expression(APP, Aβ40,42, BACE) -  brain PSN1 protein expression -  brain total-, active-GSK3β and p-Tauproteins expression - brain mRNA expression of APP -  immunoreactivity of APP and Aβ at both hippocampus and cortex - induced insolubleAβ40, and Aβ42 aggregation | Obese-insulin resistance is associated with inflammation, adipokine dyshomeostasis, increased oxidative stress, mitochondrial dysfunction and the Alzheimer’s like pathologies, leading to neurodegeneration. | (Nuzzo et al., 2015) |
| Female Wistar rats | - High-fat diet (59.28%E from fat, HFD) for 4, 8 and 12 weeks - Whole brain and hippocampal slices | **4 weeks of diet consumption**  ***HFD rats (compared with ND rats)***   -  body weight and visceral fats   **8 weeks of diet consumption**  ***HFD rats (compared with ND rats)***   -  body weight and VF -  plasma insulin, TC, LDL, HOMA index and AUCg -  glucose response in OGTT -  plasma and brain MDA -  insulin-induced LTD and LTP -  dendritic spine number   **12 weeks of diet consumption**  ***HFD rats (compared with ND rats)***   -  body weight and VF -  plasma insulin, TC, LDL, HOMA index and AUCg -  glucose response in glucose tolerance test -  plasma and brain MDA -  brain p-IR and p-AktSer473 expression -  brain mitochondrial ROS production -  brain mitochondrial depolarization -  brain mitochondrial swelling -  insulin-induced LTD and LTP -  dendritic spine number -  time to reach platform -  time spent in target quadrant | The peripheral insulin resistance developed prior to brain insulin resistance, brain mitochondrial dysfunction, hippocampal synaptic dysfunction and cognitive decline in HFD-fed female rats. | (Pratchayasakul et al., 2015) |
| C57BL/6 mice (4 weeks-old) | - High-fat diet (HFD) for 20 weeks - Whole brain | **HFD**-**fed mice**   -  body weight, insulin, glucose, FFA and TC - glucose intolerance by  glucose level on glucose challenge -  brain mitochondrial ROS production -  brain mitochondrial depolarization -  ATP content -  brain IRS-1, p-Akt, p-AMPK172 and p-GSK-3β expression -  brain p-IRS-1 expression -  RI -  escape latency -  time spent in target quadrant and crossing-target number | Long-term HFD consumption caused cognitive decline by impairment brain insulin signaling and mitochondrial dysfunction, possibly via the activation of AMPK. | (Wang et al., 2015) |
| C57BL/6 mice (2 months old) | - High-fat diet (HFD, 45%E from fat) for 5 or 13 weeks - Whole brain | **HFD**-**fed mice**  ***5 weeks***   -  body weight -  brain Mfn-2 protein expression in arcuate nucleus   ***13 weeks***   -  body weight, plasma insulin, glucose, TC, LDL and TG -  brain Mfn-2 protein expression in arcuate nucleus | Saturated lipids decreased mitofusin-2 expression, in which mitofusin-2 is an important modulator of energy compromised in the brain linked to insulin resistance during obesity. | (Diaz et al., 2015) |
| C57BL/6J mice  (3 months-old) | - High-fat diet (HFD, 60%E from fat) for 12 weeks - Whole brain and hippocampal slices | **HFD**-**fed mice**   - body weight, FPG, FINS, AUCg and HOMA-IR -  neuronal GLUT3 and GLUT4 expression -  brain p-IRS-1Y608 and p-IRS-1S307/ IRS-1 ratio -  brain p-ERK1/2/ERK1/2 ratio -  brain p-CREB/CREB ratio -  fEPSP slope and %fEPSP (LTP) | HFD could induce the impairment of brain insulin signaling and loss of synaptic strength and plasticity. | (Liu et al., 2015) |
| Male Wistar rats | - High-fat diet (59.28%E from fat, HFD) for 4, 8 and 12 weeks - Whole brain and hippocampal slices | **4 weeks of diet consumption**  ***HFD rats (compared with ND rats)***   -  body weight and VF   **8 weeks of diet consumption**  ***HFD rats (compared with NDS)***   -  body weight and VF -  plasma insulin, TC and AUCg -  plasma HDL   **12 weeks of diet consumption**  ***HFD rats (compared with ND rats)***   -  body weight and VF -  plasma insulin, TC and AUCg -  plasma HDL -  plasma and brain MDA -  brain mitochondrial ROS production -  brain mitochondrial depolarization -  brain mitochondrial swelling -  brain p-IR and p-AktSer473 proteins expression -  insulin-induced LTD -  LTP -  dendritic spine number -  time to reach platform -  time spent in target quadrant | Peripheral insulin resistance occurred prior to brain insulin resistance, brain mitochondrial dysfunction and cognitive decline in HFD-induced obesity. | (Pintana et al., 2016a) |
| Male Wistar rats | - High-fat diet (HFD, 59.28%E from fat) for 12 weeks - Whole brain and hippocampal slices | **HFD**-**fed rats**   -  body weight, insulin and HOMA index -  plasma TNF-α -  plasma and brain MDA -  brain mitochondrial ROS production -  brain mitochondrial depolarization -  brain mitochondrial swelling -  insulin-induced LTD -  dendritic spine numbers -  time to reach the platform -  time spent in target quadrant | Brain mitochondrial dysfunction, brain insulin resistance, brain apoptosis and loss of dendritic spine were observed in obese-insulin resistant rats. | (Chunchai et al., 2016) |
| Male Wistar rats | - High-fat diet (HFD, 59.28%E from fat) for 12 weeks - Whole brain and hippocampal slices | **HFD**-**fed rats**   -  body weight and VF -  plasma TC, LDL and insulin -  plasma and brain MDA -  AUCg­ and HOMA-index -  plasma HDL -  brain mitochondrial ROS production -  brain mitochondrial depolarization -  brain mitochondrial swelling -  brain p-IR and p-AktSer473 proteins expression -  insulin-induced LTD -  LTP -  dendritic spine numbers -  time to reach the platform -  time spent in target quadrant | Obese-insulin resistance impaired brain insulin signaling, brain mitochondrial dysfunction and dendritic spine loss, leading to the cognitive impairment. | (Pintana et al., 2016b) |
| Male Wistar rats | - High-fat diet (HFD, 59.28%E from fat) for 12 weeks - Whole brain and hippocampal slices | **HFD**-**fed rats**   -  body weight and VF -  plasma TC, LDL and insulin -  plasma FGF-21 -  serum and brain MDA -  serum TNF-α -  AUCg­ and HOMA-index -  plasma adiponectin and HDL -  brain mitochondrial ROS production -  brain mitochondrial depolarization -  brain mitochondrial swelling -  brain bax protein expression -  brain bcl-2 protein expression -  brain p-FGFR1, p-ERK1/2 and PGC-1α proteins expression -  LTP -  dendritic spine numbers -  time to reach the platform -  time spent in target quadrant | HFD-induced obese-insulin resistance increased plasma FGF-21 level, systemic oxidative stress-inflammation, resulting in development of cognitive impairment through brain mitochondrial dysfunction, impaired FGF-21 signaling, brain apoptosis and dendritic spine loss. | (Sa-Nguanmoo et al., 2016) |
| Male Wistar rats | - High-fat diet (HFD) consumption (59.28%E from fat) for 12 weeks - Whole brain and hippocampal slices | **HFD-fed rats**   -  body weight and VF -  plasma TC, LDL and insulin -  serum and brain MDA -  AUCg­ and HOMA-index -  brain mitochondrial ROS production -  brain mitochondrial depolarization -  brain mitochondrial swelling -  brain bax protein expression -  brain bcl-2 protein expression -  brain p-AktSer473 protein expression -  brain p-NF-κBp65/NF-κBp65 ratio -  LTP and insulin-induced LTD -  time to reach the platform -  time spent in target quadrant | Obese-insulin resistance induced by HFD consumption impaired cognitive function via brain mitochondrial dysfunction, brain apoptosis and brain inflammation-oxidative stress. | (Sa-Nguanmoo et al., 2017) |
| Sprague Dawley rats (8 weeks-old) | - Lard oil-enriched high-fat diet (HFD, 3.9 kcal/g, 34% E from fat) for 3 days - Whole brain | **HFD consumption**   - -  elongated and elliptical brain mitochondria with shorter and less branched   -  p-IRS1Ser1101 expression   -  p-Drp-1Ser637 expression   -  p-PERKT980 expression | HFD consumption induced brain mitochondrial fission, resulting induction of ER stress and insulin resistance in the brain. | (Filippi et al., 2017) |
| C57BL/6J mice (6 weeks-old) | - High-fat diet (HFD) for 12 weeks - Hippocampus | **HFD-fed mice**   -  body weight -  plasma glucose, insulin and HOMA -  p-IRSSer307 expression -  p-AktSer473 and GSK3βSer9 expression -  amyloid deposition and p-Tau expression -  SOD level and HO-1 and Nrf2 expression -  MDA, IL-1β and TNF-α levels -  escape latency -  Time spent in the target zone | HFD caused cognitive impairment by inducing brain insulin resistance, oxidative stress, inflammation and reducing Nrf2/HO-1 signaling pathway. | (FangFang et al., 2017) |
| C57BL/6NHsd mice | - High-fat diet (HFD, 40% E from fat) for 14 weeks - Whole brain | **HFD-fed mice**   -  body weightand fat mass -  serum insulin, HOMA-IR -  GTT and ITT response -  serum TC**,** HDL - plasma membrane expression of GLUT1 and GLUT3 -  expression of p-IRTyr1150/1151 -  p-IRSSer307 and p-IRSSer336/339 expression -  BACE expression -  IDE expression -  amyloid deposition, p-Tau and AMPKSer485 expression | HFD-induced brain insulin resistance, promote formation of amyloid plaques and neurofibrillary tangles. | (Kothari et al., 2017) |
| C57BL/6J mice (4 weeks-old) | - High-fat diet (HFD) for 12 weeks - Whole brain | **HFD-fed mice**   -  body weightand food intake - FPG and impaired intraperitoneal glucose tolerance (IPGT) -  serum TC, LDL and TG -  serum MDA and oxidized GSH/total GSH ratio -  serum SOD -  AChE activity -  ACh level -  p-JNK, p-IRS and p-Tau expression - alternation behavior -  latency time prior to entering the dark room -  escape latency time -  stay ratio in target area | HFD consumption triggered brain insulin resistance, impaired brain cholinergic system, reduced oxidant balance resulting in cognitive impairment. | (Kim et al., 2017) |
| Male offspring mice | - Offspring of maternal obese-mice by HFD consumption starting upon their weaning at three weeks of age - Hippocampus | **Offspring mice**   -  mTOR signaling -  neurogenesis marker expression (doublecortin) -  synaptic plasticity markers expression (ampaloxO1, p-Synapsin, vGlut and vGAT) | Maternal obesity induced offspring hippocampal insulin resistance and possibly mediate offspring’s cognitive impairment. | (Schmitz et al., 2018) |
| Male Wistar rats | - High-fat diet (HFD, 59.28%E from fat) for 12 weeks - Whole brain and hippocampal slices | **HFD**-**fed rats**   -  body weight and VF -  plasma TC, LDL and insulin -  AUCg­ and HOMA-index -  plasma adiponectin and HDL -  plasma FGF-21 -  serum and brain MDA -  plasma TNF-α -  brain mitochondrial ROS production -  brain mitochondrial depolarization -  brain mitochondrial swelling -  brain bax protein expression -  brain bcl-2 protein expression -  ratios of both p-FGFR1/total- FGFR1 and p-ERK1/2/total-ERK1/2 and PGC-1α protein expression -  LTP -  dendritic spine numbers -  time to reach the platform -  time spent in target quadrant | Long-term high-fat diet consumption induce peripheral insulin resistance, metabolic disturbances and brain pathologies, resulting in cognitive impairment. | (Sa-Nguanmoo et al., 2018) |
| **2. Genetic-induced obese-insulin resistant models** | | | | |
| Male *ob/ob* mice (14-16 weeks old) | - Whole brain and hippocampal slices | ***ob/ob* mice**   -  body weight -  non-fasting plasma glucoseand AUCg -  non-fasting plasma insulin -  glycemic responsefollowing a glucose load -  number of feeding bouts - O2 consumption, CO2 productionand respiratory exchange rate -  brain synaptophysin expression -  LTP | Obese-mice demonstrated peripheral insulin resistance and impaired brain synaptic plasticity through mediation in part through inducing mitochondrial dysfunction. | (Porter et al., 2013) |
| Elderly Zucker diabetic fatty rats (ZDF, FA/FA, 30-34 weeks old) | - Whole brain | **ZDF rats**   -  brain cellular and mitochondrial ROS -  brain NO level - brain microsomal and mitochondrial LPO, protein carbonyl content -  brain cytosolic and mitochondrial GSH -  brain catalase and mitochondrial GST, GSH-Px and GSH-reductase -  brain mitochondrial complex I-IV, ATP content -  brain p-JNK and IκB-α expression -  brain iNOS expression -  brain SOD | Diabetic brain developed the complication associated with brain redox homeostasis and brain mitochondrial dysfunction. | (Raza et al., 2015) |
| **3. Obese**-**T2DM** | | | | |
| Male Sprague Dawley rats (6 weeks old) | - T2DM induction (HFD for 4-weeks followed by 30 mg/kg of STZ, IP) - Whole brain | **Before STZ injection**   -  body weight, food intake and water intake   **After STZ injection**   -  body weight -  FSI, blood glucose of OGTT, - impaired insulin sensitivity in ITT -  brain p-IRS2Ser731 expression and amyloid-β1-42level -  p-AktSer473 and p-GSK-3βSer9 expression - dissipation of mitochondrial ΔΨm and ROS production - brain mitochondrial swelling and morphological changes -  escape latency and swimming distance -  time spent in target quadrant -  number of times for crossing the original platform | Diabetes-associated cognitive decline (DACD) may be caused by brain mitochondrial dysfunction, insulin insensitivity and histopathological change ( β-amyloid). | (Sun et al., 2016) |

*AMPK; adenosine monophosphate-activated protein kinase, APP; amyloid precursor protein, ATP; adenosine triphosphate, AUCg; area under the curve of glucose, Aβ; amyloid-beta, BACE; beta-site APP-cleaving enzyme, BW; body weight, CREB; cAMP response element-binding protein, DPP; dipeptidyl peptidase, Drp; dynamin-related protein, DVC; dorsal vagal complex, ER; endoplasmic reticulum, ERK; Extracellular signal-regulated kinase, fEPSP; field excitatory post-synaptic potential, FIS1; Mitochondrial fission 1, FFA; free fatty acid, FGF; fibroblast growth factor; FGFR; fibroblast growth factor receptor, FINS; fasting insulin, FPG; fasting plasma glucose, FPI; fasting plasma insulin, FSI; fasting serum insulin, GLP-1; glucagon-like peptide 1, GLP; glucagon-like peptide, GLUT; glucose transporter, GSH; glutathione, GSH-Px; glutathione peroxidase, GSK-3β; glycogen synthase kinase-3-beta, GST; Glutathione S-transferases, HbA1C; glycated hemoglobin, HDL; high-density lipoprotein, HOMA; homeostasis model assessment, HSP; heat shock protein, ICV; intracerebroventricular injection, IκB; I kappa B, IL; interleukin, iNOS; inducible nitric oxide synthase, IP; intraperitoneal injection, ITT; insulin tolerance test, IR; insulin receptor, IRS; insulin receptor substrate, JNK; -Jun N-terminal kinases, LDL; low-density lipoprotein, LPO; lipid peroxidation, LTD; long-term depression, LTP; long-term potentiation, MAP2; microtubule associated protein-2, MAPK; mitogen-activated protein kinase, Mash1; mammalian achaete scute homolog 1, MCP-1; monocyte chemoattractant protein-1, MDA; malondialdehyde, MDIVI; mitochondrial division inhibitor, Mfn; mitofusin, mTOR; mammalian target of rapamycin, NF-κB; nuclear-factor kappa B, NMDARs; N-methyl-D-aspartate receptors, Nrf; nuclear respiratory factor, OGTT; oral glucose tolerance test, OPA; optic atrophy protein, PERK; protein kinase-like endoplasmic reticulum kinase, PGC-1α; peroxisome proliferator-activated receptor gamma coactivator 1-alpha, PO; oral gavage feeding, PPAR-γ; peroxisome proliferator-activated receptor gamma, PSD95; post-synaptic density, PTP-1B; protein tyrosine phosphatase-1B, RI; recognition index, ROS; reactive oxygen species, SGLT; sodium-glucose cotransporter, SIRT1; silent information regulator-1, SOD; superoxide dismutase, SREBP; sterol regulatory element-binding protein, STZ; streptozotocin, TC; total cholesterol, TFAM; mitochondrial transcription factor A, TG; triglyceride, TNF-α; tumor necrosis factor-alpha, VDAC; voltage-dependent anion channel, VF; visceral fat, vGAT; vesicular GABA transporter, vGLUT; vesicular Glutamate transporter, VLDL; very-low density lipoprotein*.
