# Supplementary material for: Links Between Obesity-Induced Brain Insulin Resistance, Brain Mitochondrial Dysfunction, and Dementia
Source: Front Endocrinol (Lausanne). 2018 Aug 31;9:496. doi: 10.3389/fendo.2018.00496 (PMC6127253; doi:10.3389/fendo.2018.00496)
Supplement: Supplementary file 3 [file Table_3.DOC]

**Supplementary Table 3:** Effects of pharmacological interventions on brain dysfunction induced by obesogenic condition

| **Study Models** | **Methods** | **Major findings** | **Interpretation** | **Refs.** |
| --- | --- | --- | --- | --- |
| ***In vitro* study** | | | | |
| **Unsaturated free fatty acid** | | | | |
| Hypothalamic mHypoA-CLU192 cells | - Palmitic acid  (250 and 500 µM) exposure for 6, 12, 24 and 48 hours - 4-Phenylbutyrate (PA, 2 μM) prior to paltimitic acid exposure - Sodium salicylate (50 μM) prior to paltimitic acid exposure | **PA or Sodium salicylate pretreatment before palmitic acid**   -  p-AktSer473 protein expression -  Mfn-2 protein expression | Inhibition of inflammation or ER stress prior to sFFA exposure attenuated neuronal insulin-resistant condition. | (Diaz et al., 2015) |
| ***In vivo* study** | | | | |
| **1. Anti-diabetic drugs** | | | | |
| Male Sprague Dawley rats | - High-fat diet (HFD, 58%E from fat) for 5 weeks - Rosiglitazone (5 mg/kg), PO for 7 days | **Metformin-treated rats**   -  body weight -  plasma glucose, TC, TG and insulin -  escape latency | Rosiglitazone restores cognitive decline by attenuating peripheral insulin resistance in HFD-fed rats. | (Pathan et al., 2008) |
| Male Wistar rats | - High-fat diet (HFD, 59.28%E from fat) for 12 weeks - Rosiglitazone  (5 mg/kg), PO for 14 days - Whole brain and hippocampal slices | **Metformin-treated rats**   -  body weight and VF -  plasma glucose, TC and insulin -  AUCg­ and HOMA-index -  brain mitochondrial ROS production -  brain mitochondrial depolarization -  brain mitochondrial swelling -  brain p-AktSer473 expression -  insulin-induced LTD | Neuronal insulin resistance and the impairment of brain mitochondria caused by a 12-wk HFD consumption can be reversed by rosiglitazone. | (Pipatpiboon et al., 2012) |
| Male Wistar rats | - High-fat diet (HFD, 59.28%E from fat) for 12 weeks - Metformin  (15 mg/kg, twice daily), PO for 21 days - Whole brain | **Metformin-treated rats**   -  body weight and VF -  plasma TC and insulin -  plasma and brain MDA -  AUCg­ and HOMA-index -  brain mitochondrial ROS production -  brain mitochondrial depolarization -  brain mitochondrial swelling -  time to reach the platform -  time spent in target quadrant | Metformin effectively improves peripheral insulin sensitivity, prevents  brain mitochondrial dysfunction, and completely restores learning behavior, which were all impaired by  long-term HFD consumption. | (Pintana et al., 2012) |
| Male Wistar rats | - High-fat diet (HFD, 59.28%E from fat) for 12 weeks - Vildagliptin (DPP-4 inhibitor,  3 mg/kg), PO for 21 days - Whole brain and hippocampal slices | **Vildagliptin-treated rats**   -  body weight and VF -  plasma TC and insulin -  plasma and brain MDA -  AUCg­ and HOMA-index -  plasma and brain GLP-1 -  k-value -  brain mitochondrial ROS production -  brain mitochondrial depolarization -  brain mitochondrial swelling -  brain p-AktSer473, p-IR and p-IRS proteins expression -  insulin-induced LTD -  time to reach the platform -  time spent in target quadrant | Vildagliptin effectively restored neuronal IR function, increased glucagon-like-peptide 1 levels and prevented brain mitochondrial  dysfunction, thus attenuating the impaired cognitive function caused by HFD. | (Pipatpiboon et al., 2013) |
| Male Wistar rats | - High-fat diet (HFD, 59.28%E from fat) for 12 weeks - Vildagliptin (DPP-4 inhibitor, 3 mg/kg), PO for 4 weeks - Sitagliptin (DPP-4 inhibitor, 30 mg/kg), PO for 21 days - Whole brain | **Vildagliptin or sitagliptin-treated rats**   -  body weight -  VF -  plasma TC and insulin -  plasma and brain MDA -  AUCg­, AUCi and HOMA-index -  plasma GLP-1 and HDL -  brain mitochondrial ROS production -  brain mitochondrial depolarization -  brain mitochondrial swelling -  time to reach the platform -  time spent in target quadrant | Inhibition DPP-4 enzyme with vildagliptin or sitagliptin increases peripheral insulin sensitivity and decreases brain dysfunction in insulin-resistant rats | (Pintana et al., 2013) |
| Male NIH Swiss mice (6 to 8 weeks-old) | - High-fat diet (HFD, 45%E from fat) for 12 weeks - Metformin (300 mg/kg), PO for 20 days - Whole brain and hippocampal slices | **Metformin-treated mice**   -  body weight, energy intake and non-fasting glucose and insulinlevel -  glucose-stimulated plasma insulin concentrations -  IRS-1, mTOR, NTRK2, SIRT1 and VEGF hippocampal genes expression - LTP -  RI | Metformin treatment decreased cognitive decline in HFD-fed mice by elevating synaptic plasticity and cognitive-related gene expression. | (Lennox et al., 2014b) |
| Male Wistar rats | - High-fat diet (HFD, 59.28%E from fat) for 12 weeks - Vildagliptin (DPP-4 inhibitor, 3 mg/kg), PO for 4 weeks - Pioglitazone (PPARγ agonist,10 mg/kg), PO for 3 weeks - Whole brain and hippocampal slices | **Vildagliptin-treated rats**   -  plasma TC and HOMA-index -  dendritic spine numbers   **Pioglitazone-treated rats**   -  body weight and VF -  plasma TC, glucose, insulin and HOMA-index -  dendritic spine numbers | DPP-4 inhibitor and PPARγ agonist restored the reduction of dendritic spines caused by HFD, suggesting the beneficial roles of DPP-4 inhibitors and PPARγ agonists in neurodegenerative disorders. | (Sripetchwandee et al., 2014) |
| Male NIH/OlaHsd mice (6 to 8weeks-old) | - High-fat diet (HFD) for 20 weeks - Sitagliptin (DPP-4 inhibitor, 50 mg/kg), PO for 21 days - Whole brain and hippocampal tissues | **Sitagliptin-treated mice**   -  body weight and energy intake -  non-fasting glucose, glucose concentration after glucose loading, AUCg and HOMA-IR -  plasma TG and TC -  plasma insulin, AUC of insulin and HOMA-β -  plasma DPP-4 activity -  plasma GLP-1, brain GLP-1 and brain GIP activities -  hippocampal genes expression of GLP-1 receptor, GIPR, synaptophysin, SIRT1, GSK-3β, SOD2, Nrf2 and VEGF -  number of DCX-positive cells in dentate gyrus -  hippocampal 8-oxoguanine-positive cells -  RI | Sitagliptin restore memory function in HFD-fed mice via improving insulin  sensitivity, enhancing hippocampal neurogenesis, and reducing oxidative stress. | (Gault et al., 2015) |
| Male Wistar rats | - High-fat diet (HFD, 59.28%E from fat) for 12 weeks - A restricted diet (normal diet, containing only 60% energy intake) - Vildagliptin (DPP-4 inhibitor, 3 mg/kg), PO for 4 weeks - Whole brain and hippocampal slices | **Combined a restricted diet-fed rats with vildagliptin**   -  body weight and VF -  plasma TC, LDL and insulin -  plasma and brain MDA -  AUCg­ and HOMA-index -  plasma HDL -  brain mitochondrial ROS production -  brain mitochondrial depolarization -  brain mitochondrial swelling -  brain p-IR and p-AktSer473 proteins expression -  LTP and insulin-induced LTD -  dendritic spine numbers -  time to reach the platform -  time spent in target quadrant | Combination of energy restriction and DPP-4 inhibitor provide neuroprotection in obese-insulin resistant male rats. | (Pintana et al., 2016b) |
| C57BL/6J mice (6 weeks-old) | - High-fat diet (HFD) for 12 weeks - Metformin (200 mg/kg), PO for 12 weeks - Hippocampus | **Metformin-treated mice**   -  body weight -  plasma glucose, insulin and HOMA -  p-IRSSer307 expression -  p-AktSer473 and GSK3βSer9 expression -  amyloid deposition and p-Tau expression -  SOD level and HO-1 expression -  MDA, IL-1β and TNF-α levels -  escape latency -  Time spent in the target zone | Metformin ameliorates cognitive impairment in HFD-fed mice via an improvement of peripheral insulin sensitivity, brain insulin sensitivity and an attenuation of oxidative stress, inflammation. | (FangFang et al., 2017) |
| Male Wistar rats | - High-fat diet (HFD) consumption (59.28%E from fat) for 12 weeks - Vildagliptin (DPP-4 inhibitor, 3 mg/kg), PO for 4 weeks - Dapaglifozin (SGLT-2 inhibitor, 1 mg/kg), PO for 4 weeks - Whole brain and hippocampal slices | **Vildagliptin-treated rats**   -  plasma TC, LDL and insulin -  serum and brain MDA -  AUCg­ and HOMA-index -  brain mitochondrial ROS production -  brain mitochondrial depolarization -  brain mitochondrial swelling -  bax protein expression -  bcl-2 protein expression -  p-AktSer473 protein expression -  p-NF-κBp65/NF-κBp65 ratio -  LTP -  insulin-induced LTD -  time to reach the platform -  time spent in target quadrant   **Dapaglifozin-treated rats**   -  body weight and VF -  plasma TC, LDL and insulin -  glucose urinary excretion (UGE) -  serum and brain MDA -  AUCg­ and HOMA-index -  brain mitochondrial ROS production -  brain mitochondrial depolarization -  brain mitochondrial swelling -  brain bax protein expression -  brain bcl-2 protein expression -  brain p-AktSer473 protein expression -  brain p-NF-κBp65/NF-κBp65 ratio -  LTP -  insulin-induced LTD -  time to reach the platform -  time spent in target quadrant   **Combined dapaglifozin and vildagliptin-treated rats**   -  body weight and VF -  plasma TC, LDL and insulin -  glucose urinary excretion (UGE) -  serum and brain MDA -  AUCg­ and HOMA-index -  brain mitochondrial ROS production -  brain mitochondrial depolarization -  brain mitochondrial swelling -  brain bax protein expression -  brain bcl-2 protein expression -  brain p-AktSer473 protein expression -  brain p-NF-κBp65/NF-κBp65 ratio -  LTP -  insulin-induced LTD -  time to reach the platform -  time spent in target quadrant | Combined SGLT-2 inhibitor with DPP-4 inhibitor could be the best approach for neuroprotection in obese-insulin resistance. | (Sa-Nguanmoo et al., 2017) |
| Male Wistar rats | - High-fat diet (HFD, 59.28%E from fat) for 12 weeks - vildagliptin (DPP-4 inhibitor, 3 mg/kg), PO for 4 weeks - Whole brain and hippocampal slices | **Vildagliptin-treated rats**   -  body weight and VF -  plasma TNF-α -  plasma TC, LDL and insulin -  AUCg­ and HOMA-index -  plasma adiponectin and HDL -  plasma FGF-21 -  serum and brain MDA -  brain mitochondrial ROS production -  brain mitochondrial depolarization -  brain mitochondrial swelling -  brain bax protein expression -  brain bcl-2 protein expression -  ratios of both p-FGFR1/total- FGFR1 and p-ERK1/2/total-ERK1/2 -  PGC-1α protein expression -  LTP -  dendritic spine numbers -  time to reach the platform -  time spent in target quadrant | Vildagliptin ameliorates cognitive impairment in HFD-fed rats via reducting peripheral insulin resistance and systemic inflammation, attenuating brain apoptosis, and restoring brain mitochondrial function and dendritic spine density. | (Sa-Nguanmoo et al., 2018) |
| **2. Hormone therapy (incretin and fibroblast growth factor-21)** | | | | |
| Male Swiss TO mice (6–8 weeks old) | - High-fat diet (HFD, 45%E from fat) for 8 months - exendin-4 (incretin mimetic drug, 25 nmol/kg), SC for 21 days - Whole brain and hippocampal slices | **Exendin-4**-**treated mice**   - body weight -  average energy intake, plasma glucose and AUCg -  plasma insulinand AUCi -  LTP - RI in trial phase | Exendin-4 therapy improves cognitive function and ameliorates impaired hippocampal synaptic plasticity in dietary-induced obesity. | (Gault et al., 2010) |
| Male Swiss TO mice (6 to 8 weeks-old) | - High-fat diet (HFD, 45%E from fat) for 20 weeks - Liraglutide (incretin mimetic drug, 200 μg/kg), SC for 28 days - Whole brain and hippocampal slices | **Liraglutide-treated mice**   - body weight gain, energy intake, non-FPG and AUCg -  non-FPI - improved glucose response - LTP - motor activity,number of grooming and rearing - RI in trial phase | Liraglutide administered peripherally not only improves metabolic parameters but exerts additional beneficial effects on cognitive  function and hippocampal synaptic plasticity. | (Porter et al., 2010) |
| Male *ob/ob* mice (14 to 16 weeks old) | - Liraglutide (incretin mimetic drug, 50 nmol/kg, twice daily), SC for 21 days - Whole brain and hippocampal slices | **Liraglutide-treated mice**   -  body weight -  non-FPGand AUCg -  non-FPI -  glycemic responsefollowing a glucose load -  number of feeding bouts -  brain Mash1 -  brain synaptophysin expression -  LTP | Liraglutide elicits beneficial effects on metabolic control and synaptic plasticity in mice with severe obesity and insulin resistance mediated in part through increased expression of Mash1 believed to improve hippocampal neurogenesis and cell  survival. | (Porter et al., 2013) |
| Male lean mice | - High-fat diet (HFD, 45%E from fat) for 4 months - Lixisenatide (incretin-mimetic drug, 50 nmol/kg), SC for 40 days - Hippocampal slices | **Lixisenatide-treated mice**   -  body weight -  plasma glucose, AUCg and HOMA-IR -  plasma insulin and HOMA-β -  BrdU-positive cells in the dentate gyrus -  hippocampal mTOR and NTRK2 gene expression -  RI | Lixisenatide improves metabolic regulation and enhance cognitive function in obese-model. | (Lennox et al., 2014a) |
| Male Wistar rats | - High-fat diet (HFD, 59.28%E from fat) for 12 weeks - Recombinant human FGF-21 (0.1 mg/kg), IP for 28 days - Whole brain and hippocampal slices | **FGF-21-treated rats**   -  body weight and VF -  plasma TC, LDL and insulin -  plasma FGF-21 -  serum and brain MDA -  serum TNF-α -  AUCg­ and HOMA-index -  plasma adiponectin and HDL -  brain mitochondrial ROS production -  brain mitochondrial depolarization -  brain mitochondrial swelling -  bax protein expression -  bcl-2 protein expression -  p-FGFR1, p-ERK1/2 and PGC-1α proteins expression -  LTP -  dendritic spine numbers -  time to reach the platform -  time spent in target quadrant | FGF-21 exerts neuroprotection in obese-insulin resistance. | (Sa-Nguanmoo et al., 2016) |
| Male Wistar rats | - High-fat diet (HFD, 59.28%E from fat) for 12 weeks - recombinant human FGF21 (0.1 mg/kg), IP for 4 weeks - Whole brain and hippocampal slices | **FGF-treated rats**   -  body weight and VF -  plasma TC, LDL and insulin -  AUCg­ and HOMA-index -  plasma adiponectin and HDL -  plasma FGF-21 -  serum and brain MDA -  plasma TNF-α -  brain mitochondrial ROS production -  brain mitochondrial depolarization -  brain mitochondrial swelling -  brain bax protein expression -  brain bcl-2 protein expression -  ratios of both p-FGFR1/total- FGFR1 and p-ERK1/2/total-ERK1/2 and PGC-1α protein expression -  LTP -  dendritic spine numbers -  time to reach the platform -  time spent in target quadrant | FGF-21 improve cognitive function in HFD-fed rats by an attenuation of peripheral insulin resistance, oxidative stress, brain mitochondrial dysfunction, hippocampal synaptic dysplasticity, dendritic spine loss and brain apoptosis. | (Sa-Nguanmoo et al., 2018) |
| Male Swiss *TO* mice (6-8 weeks old) | - High-fat diet (HFD, 45%E from fat) for 4 months - Incretin metabolites (GLP-1(9-36), GIP (3-42) and exendin (9-39),  (each of 25 nmol/kg), SC for 60 days - Whole brain and hippocampal slices | **Incretin-treated mice (compared with HFD mice)**   -  body weight, non-FPG, impaired glucose and insulin response -  O2 consumption or CO2 production -  locomotor activity - LTP -  RI on trial phase | Incretin metabolites do not influence locomotor activity, cognitive function and hippocampal synaptic plasticity, when administered at pharmacological doses to mice fed a high-fat diet. | (Porter et al., 2012) |
| **3. Herbal medicine** | | | | |
| Male B6.V-Lepob/J mice on a C57BL/6J background (ob/ob) (6-weeks old) | - High fat diet (HFD, 45%E from fat) for 6 weeks - Cinnamon extract (0.8 g/kg) in drinking water (4.5 ml/kg) for 6 weeks - Whole brain | **Cinnamon-treated mice (compared with HFD-fed mice)**   -  body weight, body fat mass, FPG, HOMA-IR, AUCg and insulin secretion after the glucose load -  PPAR-γ, SREBP, MCP-1, IL-6, IL-1β, SIRT1 genes expression in fat tissue -  energy expenditure and respiratory quotient -  brain p-AktSer473 and p-IR expression -  theta frequency band -  random locomotor activity during day and night -  locomotor activity after insulin ICV stimulation -  insulin-stimulated PTP-1B expression | Cinnamon extract improved insulin action in the brain as well as brain activity and locomotion. | (Sartorius et al., 2014) |
| Male Wistar rats | - High-fat diet (HFD, 59.28%E from fat) for 12 weeks - Garlic (250 and 500 mg/kg), PO for 28 days - Whole brain | **Garlic 250 or 500 mg/kg treatment**   -  body weight -  VF, plasma TC and insulin -  plasma and brain MDA -  AUCg­ and HOMA-index -  brain mitochondrial ROS production -  brain mitochondrial depolarization -  brain mitochondrial swelling -  time to reach the platform -  time spent in target quadrant | Garlic extract improves cognitive function through attenuating peripheral insulin resistance, oxidative and increasing brain mitochondrial function | (Pintana et al., 2014) |
| C57BL/6 mice (4-weeks old) | - High-fat diet (HFD) for 20 weeks - Naringin (100 mg/kg) in either normal chow or HFD - Whole brain | **Naringin-treated mice**   -  body weight, insulin, glucose, free fatty acid and cholesterol -  glucose intolerance by  glucose level on glucose challenge -  brain mitochondrial ROS production -  brain mitochondrial depolarization -  ATP content -  brain IRS-1, p-Akt, p-AMPK172 and p-GSK-3β expression -  brain p-IRS-1 expression -  RI -  escape latency -  time spent in target quadrant and crossing-target number | Enhancement in insulin signaling and a decrease in mitochondrial dysfunction through activation of AMPK is one of mechanism that naringin improves cognition in obese-mice. | (Wang et al., 2015) |
| Male Sprague Dawley rats (6-weeks old) | - T2DM induction (HFD for 4-weeks followed by 30 mg/kg of STZ, IP) - ZiBu PiYin Recipe (ZBPYR), PO for 11 weeks - Whole brain | **ZBPYR treatment**   -  blood glucose -  food intake and water intake -  brain p-IRS2Ser731 expression and Aβ1-42level -  brain p-AktSer473 and p-GSK-3βSer9 expression -  dissipation of mitochondrial membrane potential and ROS production -  brain mitochondrial swelling and morphological changes -  brain MAP2 expression -  brain cytosolic cytochrome C -  mitochondrial cytochrome C -  escape latency and swimming distance -  time spent in target quadrant -  number of time for crossing the original platform | ZBPYR provide protective effects against diabetes-associated cognitive decline (DACD) via ameliorating mitochondrial dysfunction, insulin sensitivity and histopathological change. | (Sun et al., 2016) |
| C57BL/6J mice (6 weeks-old) | - High-fat diet (HFD) for 12 weeks - Thymol (20, 40 mg/kg), PO for 12 weeks - Hippocampus | **Thymol-treated mice**   -  body weight -  plasma glucose, insulin and HOMA -  p-IRSSer307 expression -  p-AktSer473 and GSK3βSer9 expression -  amyloid deposition and p-Tau expression -  SOD level and HO-1 and Nrf2 expression -  MDA, IL-1β and TNF-α levels -  escape latency -  Time spent in the target zone | Thymol administration alleviated HFD induced cognitive impairment by improving brain insulin sensitivity, attenuating oxidative stress, inflammation and enhancing Nrf2/HO-1 signaling response. | (FangFang et al., 2017) |
| C57BL/6J mice (4 weeks-old) | - High-fat diet (HFD) for 12 weeks - Ginsenoside Re (5, 10 and 20 mg/kg), PO for 4 weeks - Whole brain | **Ginsenoside Re-treated mice (compared with HFD-fed mice)**   -  body weightand food intake - FPG and impaired intraperitoneal glucose tolerance (IPGT) -  serum TC, LDL and TG -  serum MDA and oxidized GSH/total GSH ratio -  serum SOD -  AChE activity -  ACh level -  p-JNK, p-IRS and p-Tau expression - alternation behavior -  latency time prior to entering the dark room -  escape latency time -  stay ratio in target area | Ginsenoside Re attenuated cognitive impairment in HFD-fed mice by reducing brain insulin resistance, preserving cholinergic and antioxidant system. | (Kim et al., 2017) |
| **4. Subcellular targeting intervention; NMDARs antagonist and mitochondrial modulator** | | | | |
| *db/db*-transgenic mice (1ขmonth old) | - 0.01% Memantine (NMDARs antagonist, 1 mg/kg), twice daily, IP for 3 months - Whole brain | ***db/db* mice treated with memantine**   - dendritic branchingand intersections - restoredspine density | NMDARs antagonist rescued dendritic arborization and synaptic density in T2DM mouse model. | (Akhtar et al., 2016) |
| 8-weeks old Sprague Dawley rats | - Lard oil-enriched high-fat diet (HFD, 3.9 kcal/g, 34% fat) for 3 days - 100 µM, 2 µl, MDIVI-1 (mitochondrial fission inhibitor), DVC infusion - A FLAG-tagged dominant-negative form of Drp-1 (Drp-1-KA), DVC infusion - A FLAG-tagged phosphor-deficient mutant of Drp-1 (Drp-1-SA), DVC infusion - 5.4 µg/µl of 4-phenylbutylrate (4-PBA), ER stress inhibitor, DVC infusion - Whole brain | **MDIVI-1 treatment with insulin co-infusion**   - reverse changes of brain mitochondrial morphology - restore DVC insulin to regulate glucose kinetics and lower glucose production   -  brain p-IRS1Ser1101 expression -  brain p-Drp-1Ser637 expression - failed to reduce brain p-PERKT980 expression -  iNOS expression   **Drp-1-KA injection (Drp-1 molecular inhibition) with insulin co-infusion**   -  number of mitochondria -  mitochondria in aspect ratio - restore ability of DVC insulin to increase glucose infusion rate and lower glucose production   **Drp-1-SA injection (Drp-1 molecular activation) with insulin co-infusion**   -  mitochondrial number -  mitochondria in aspect ratio - DVC insulin failed to alter glucose kinetics -  brain iNOS expression   **4-BPA treatment with insulin co-infusion**   - Restore ability of DVC insulin to increase glucose infusion rate and lower glucose production   -  brain p-PERKT980 expression   - failed to reduce brain Drp-1 phosphorylation | Inhibition of Drp-1 dependent mitochondrial fission in DVC attenuates insulin resistance through prevention of ER stress and iNOS expression. | (Filippi et al., 2017) |
| **5. Non-pharmacological intervention; energy restriction and vagus nerve stimulation** | | | | |
| Male Wistar rats | - High-fat diet (HFD, 59.28%E from fat) for 12 weeks - A restricted diet (normal diet, containing only 60% energy intake) - Whole brain and hippocampal slices | **Restricted diet-fed rats (compared with HFD-fed rats)**   -  body weight and VF -  plasma TC, LDL and insulin -  plasma and brain MDA -  AUCg­ and HOMA-index -  plasma HDL -  brain mitochondrial ROS production -  brain mitochondrial depolarization -  brain mitochondrial swelling -  brain p-IR and p-AktSer473 proteins expression -  LTP and insulin-induced LTD -  dendritic spine numbers -  time to reach the platform -  time spent in target quadrant | Energy restriction attenuate metabolic disturbance leading to preserve dendritic density in obese-insulin resistant rats. | (Pintana et al., 2016b) |
| Male Wistar rats | - High-fat diet (HFD, 59.28%E from fat) for 12 weeks - Vagus Nerve Stimulation (VNS, a continuous 14 s delivery of stimulation at a frequency of 20 Hz, pulse width of 500μs and a current of 0.5–0.75 mA followed by a 48 second rest) - Whole brain and hippocampal slices | **VNS treatment (compared with HFD rats)**   -  body weight and VF -  plasma insulin, TC, TG and LDL/VLDL ratio -  plasma and brain TNF-α -  plasma and brain MDA -  AUCg­ and HOMA-index -  brain mitochondrial ROS production -  brain mitochondrial depolarization -  brain mitochondrial swelling -  brain p-IR expression -  brain bax expression and bax/bcl-2 ratio -  insulin-induced LTD -  dendritic spine numbers -  time to reach the platform -  time spent in target quadrant | VNS attenuates cognitive decline in obese-insulin resistance rats by attenuating brain mitochondrial dysfunction, brain insulin resistance, brain apoptosis and increasing dendritic spine. | (Chunchai et al., 2016) |

*AMPK; adenosine monophosphate-activated protein kinase, ATP; adenosine triphosphate, AUCg; area under the curve of glucose, Aβ; amyloid-beta, DPP; dipeptidyl peptidase, Drp; dynamin-related protein, DVC; dorsal vagal complex, ER; endoplasmic reticulum, ERK; Extracellular signal-regulated kinase, FIS1; Mitochondrial fission 1, FFA; free fatty acid, FGF; fibroblast growth factor; FGFR; fibroblast growth factor receptor, FPG; fasting plasma glucose, FPI; fasting plasma insulin, GIPR; gastric inhibitory polypeptide receptor, GLP-1; glucagon-like peptide 1, GLP; glucagon-like peptide, GSK-3β; glycogen synthase kinase-3-beta, HbA1C; glycated hemoglobin, HOMA; homeostasis model assessment, ICV; intracerebroventricular injection, IκB; I kappa B, IL; interleukin, iNOS; inducible nitric oxide synthase, IP; intraperitoneal injection, IR; insulin receptor, IRS; insulin receptor substrate, JNK; -Jun N-terminal kinases, LDL; low-density lipoprotein, LTD; long-term depression, LTP; long-term potentiation, MAP2; microtubule associated protein-2, MAPK; mitogen-activated protein kinase, Mash1; mammalian achaete scute homolog 1, MCP-1; monocyte chemoattractant protein-1, MDA; malondialdehyde, MDIVI; mitochondrial division inhibitor, Mfn; mitofusin, mTOR; mammalian target of rapamycin, NF-κB; nuclear-factor kappa B, NMDARs; N-methyl-D-aspartate receptors, Nrf; nuclear respiratory factor, OPA; optic atrophy protein, PERK; protein kinase-like endoplasmic reticulum kinase, PGC-1α; peroxisome proliferator-activated receptor gamma coactivator 1-alpha, PO; oral gavage feeding, PPAR-γ; peroxisome proliferator-activated receptor gamma, PSD95; post-synaptic density, PTP-1B; protein tyrosine phosphatase-1B, RI; recognition index, ROS; reactive oxygen species, SGLT; sodium-glucose cotransporter, SIRT1; silent information regulator-1, SOD; superoxide dismutase, SREBP; sterol regulatory element-binding protein, TC; total cholesterol, TFAM; mitochondrial transcription factor A, TG; triglyceride, TNF-α; tumor necrosis factor-alpha, VEGF; vascular endothelial growth factor, VF; visceral fat, VLDL; very-low density lipoprotein*.
